# The evolution of domain-content in bacterial genomes

**DOI:** 10.1186/1745-6150-3-51

**Published:** 2008-12-11

**Authors:** Nacho Molina, Erik van Nimwegen

**Affiliations:** 1 Biozentrum, University of Basel, and Swiss Institute of Bioinformatics Klingelbergstrasse 50/70, 4056-CH, Basel, Switzerland

## Abstract

**Background:**

Across all sequenced bacterial genomes, the number of domains *n*_*c *_in different functional categories *c *scales as a power-law in the total number of domains *n*, i.e. $n_{c}\propto n^{\alpha_{c}}$, with exponents *α*_*c *_that vary across functional categories. Here we investigate the implications of these scaling laws for the evolution of domain-content in bacterial genomes and derive the simplest evolutionary model consistent with these scaling laws.

**Results:**

We show that, using only an assumption of time invariance, the scaling laws uniquely determine the relative rates of domain additions and deletions across all functional categories and evolutionary lineages. In particular, the model predicts that the rate of additions and deletions of domains of category *c *is proportional to the number of domains *n*_*c *_currently in the genome and we discuss the implications of this observation for the role of horizontal transfer in genome evolution. Second, in addition to being proportional to *n*_*c*_, the rate of additions and deletions of domains of category *c *is proportional to a category-dependent constant *ρ*_*c*_, which is the same for all evolutionary lineages. This 'evolutionary potential' *ρ*_*c *_represents the relative probability for additions/deletions of domains of category *c *to be fixed in the population by selection and is predicted to equal the scaling exponent *α*_*c*_. By comparing the domain content of 93 pairs of closely-related genomes from all over the phylogenetic tree of bacteria, we demonstrate that the model's predictions are supported by available genome-sequence data.

**Conclusion:**

Our results establish a direct quantitative connection between the scaling of domain numbers with genome size, and the rate of addition and deletions of domains during short evolutionary time intervals.

**Reviewers:**

This article was reviewed by Eugene V. Koonin, Martijn A. Huynen, and Sergei Maslov.

## Background

When the first gene sequences became available in the 1960s some striking and unexpected patterns were observed. For example, comparison of the fossil record with the number of amino acid substitutions separating orthologous proteins in mammals [1] suggested a constant rate of amino acid substitutions. In addition, the inferred rate of amino acid substitutions was so high that it was hard to imagine how all of these substitutions could have been fixed by the action of natural selection [2]. This famously lead Kimura to propose the neutral theory of molecular evolution [3]. Neutral evolution became the *de facto *null model of sequence evolution and the availability of such a null model was crucial for the development of rigorous methods for reconstructing evolutionary phylogenies (e.g. [4]) and methods for detecting selection acting on gene sequences (e.g. [5,6]).

Evolution of course also takes place at higher levels of organization than substitutions within protein-coding genes. In particular, large genomic segments containing one or more genes can be duplicated or deleted, and segments can be 'horizontally transfered', i.e. taken from one organism's genome and inserted into another organism's genome. Through such events organisms can vary the gene content of their genomes, acquiring genes with new functions, sub-functionalizing existing functions, or deleting genes whose functions are no longer required. Now that the sequences of several hundred of whole microbial genomes have become available over the last decade it has become possible to investigate variation in gene-content across genomes in a quantitative manner.

Studies of gene content have uncovered several striking quantitative 'laws'. First of all, it was noticed [7-9] that a number of key genomic quantities show power-law distributions. In particular, the distribution of gene family sizes is a power-law in each genome, whose exponent appears to depend mostly on the size of the genome. Several theoretical models have been put forward for explaining these power-law distributions, which all include gene duplications and deletions as key ingredients. Another striking observation [10] is that the numbers of genes in different functional categories scale as power-laws in the total number of genes in the genome. For example, whereas the numbers of genes involved in different types of metabolism scale approximately linear with genome size, the number of genes involved with regulatory processes such as transcription regulation and signal transduction scales roughly quadratically with genome size, and the number of genes involved with basic processes such as DNA replication or cell division scales with an exponent less than 1. Such scaling laws are observed for the large majority of high-level functional categories of genes and appear to apply to all bacterial genomes.

As we have argued previously [10,11], these scaling laws have important implications for the evolutionary dynamics of gene duplications and deletions and we will here investigate these implications in detail. The organization of the paper is as follows. We study genome evolution at the level of protein domains and we start by demonstrating that scaling laws are also observed at the level of the number of protein-domains.

We re-estimate the scaling exponents *α*_*c *_using all 630 currently available genomes. Next, using the assumption that the scaling laws are time invariant, we derive a 'null model' for genome evolution that accounts for the observed scaling laws. In this model the exponents of the scaling laws are identified as universal constants of the evolutionary process.

We collected 93 pairs of closely-related bacterial genomes and tested the model's predictions by analyzing the protein-domain content of these genomes and estimating, for each pair, the rates at which additions and deletions of domains from different categories have occurred since their common ancestor. We show that essentially all of the model's predictions are supported by the available genome data. Finally, we also discuss the important implications of our results for the role of horizontal gene transfer in genome evolution.

## Results and Discussion

### Scaling laws in protein domain occurrences

Although genes are natural units in genome analysis there are some disadvantages to using genes as the central units in the analysis of the evolution of genome content. For example, apart from being able to mutate, duplicate, and be deleted, it is well-known that, not infrequently, two genes can fuse into one, single genes can split into two [12], and genes can evolve *de novo *from non-coding sequence. Such events significantly complicate the analysis of the evolution of gene content.

Protein domains form more natural units for the study of the evolution of gene-content for several reasons. It can be argued that protein domains act like 'evolutionary atoms' to a certain extent [9]; Protein domains form functional units [13] that cannot be split into smaller units, and a single protein domain can, in general, not be constructed by fusing multiple occurrences of other protein domains. Therefore, we can safely assume that almost all changes in the number of occurrences in the genome of a given protein domain are due to deletions, duplications, or the horizontal transfer of a domain from another organism's genome. We thus decided to study the evolution of functional gene content in terms of the number of occurrences of different protein domains. Among databases of protein domains Pfam [14] is attractive because the Pfam domain families are disjoint, i.e. at the default settings it is guaranteed that any given DNA sequence segment will be classified to belong to at most one domain family. We thus used Pfam domains as our evolutionary 'atoms'.

We counted the number of occurrences of each Pfam domain in each fully sequenced bacterial genome (Methods). Using a mapping from Pfam to Gene Ontology categories [15] we determined, for each genome *g*, the total number of domains *n*(*g*) that can be associated with *any *GO category and, for each GO category *c*, the number of domains *n*_*c*_(*g*) occurring in the genome.

**Figure 1:**
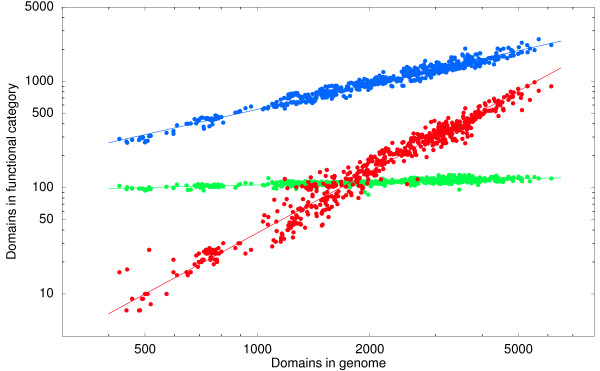
**Scaling laws**. The number of protein-domains associated with functional categories 'translation' (green), metabolic process' (blue), and 'regulation of transcription' (red) as a function of the total number of domains in the genome for which a functional annotation is available. Each dot corresponds to a fully-sequenced microbial genome, with the total number of domains on the horizontal axis and the number of domains in a particular functional category on the vertical axis. Both axes are shown on a logarithmic scale. The straight lines show power-law fits.

Figure 1 shows, for 3 example categories, the number of domains in that category as a function of the total number of domains in the genome (that can be mapped to a GO category). As the figure shows, for all three categories the number of genes in the category *n*_*c *_scales as a power-law in the total number of domains in the genome *n*, i.e.

(1) $$n_{c}=e^{\beta_{c}}n^{\alpha_{c}},$$

with both the pre-factors *β*_*c *_and the exponents *α*_*c *_varying between categories. These power-laws are observed for the large majority of high-level functional categories. For each GO category we fitted a power-law of the form (1) using a Bayesian procedure which in particular provides a posterior probability distribution for the exponent *α*_*c *_(Methods). We selected 156 GO categories that occur in at least 95% of all genomes and that show good power-law fits (Methods). The inferred exponents match what we found previously based on the gene-number analysis of a much smaller number of genomes [10,11], i.e. for basic processes such as translation and DNA repair exponents are low, whereas exponents for regulatory functions such a regulation of transcription and signal transduction are largest. The inferred exponents for all 156 selected categories are listed in Additional file 1.

### Evolutionary Model

We want to investigate the implications of the scaling laws (1) for evolutionary dynamics. That is, we want to infer what the scaling laws imply for the behavior of the domain number counts *n*_*c*_(*t*) as a function of time *t*. It is important to define precisely what we mean by *n*_*c*_(*t*). A sequenced genome *g *represents a particular bacterial strain and can idealistically be thought of as representing the genome of a single bacterial organism living today with domain counts *n*_*c*_(*g*). Since bacteria reproduce clonally we can imagine tracing this individual back through time, back to its mother cell, its grandmother, and eventually all the way back until the common ancestor of all currently sequenced genomes. We denote by *n*_*c*_(*g*, *t*) the number of domains of category *c *that were present in the ancestor organism of genome *g *that was living at time *t*.

Let *t*_now _denote today and let *x*_*c*_(*g*, *t*) denote the logarithm of the domain-number, i.e. *x*_*c*_(*g*, *t*) = log[*n*_*c*_(*g*, *t*)], and similarly *x*(*g*, *t*) = log[*n*(*g*, *t*)]. In these variables the scaling laws are just straight lines, i.e all genomes *g *(approximately) obey the linear relation

(2) *x*_*c*_(*g*, *t*_today_) = *α*_*c*_*x*(*g*, *t*_today_) + *β*_*c *_∀_*g*_.

We will now derive how these scaling laws constrain the changes in domain-numbers that have occurred throughout time. Let *t *= 0 denote the time at which the last common ancestor of all sequenced bacterial genomes was alive. Note that, since the GO categories that we consider occur in almost all genomes, it is reasonable to assume that they all had nonzero count in the last common ancestor. We let *x*_*c*_(0) denote the log-domain counts in this common ancestor and *x*(0) the logarithm of the total domain count. Further, we denote by *dx*_*c*_(*g*, *t*) the change in the log domain-count for category *c*, that occurred in a small interval of time centered around time *t *in the evolutionary history of genome *g*. The log domain-counts *x*_*c*_(*g*, *t*) and *x*(*g*, *t*) are then by definition given by the integrals

(3) $$x_{c}(g,t_{\text{now}})=x_{c}(0)+\int_{0}^{t_{\text{now}}}dx_{c}(g,t),$$

and

(4) $$x(g,t_{\text{now}})=x(0)+\int_{0}^{t_{\text{now}}}dx(g,t).$$

Comparing equations (3) and (4) with equation (2) the scaling laws thus imply that we have

(5) $$x_{c}(0)+\int_{0}^{t_{\text{now}}}dx_{c}(g,t)=\beta_{c}+\alpha_{c}\left[x(0)+\int_{0}^{t_{\text{now}}}dx(g,t)\right]\forall g.$$

Since (5) must hold for *all *genomes *g*, this equation first of all implies a relation between the offsets *β*_*c *_and the domain counts in the last common ancestor:

(6) *β*_*c *_= *x*_*c*_(0) - *α*_*c*_*x*(0).

More importantly, we find that all genomes must obey

(7) $$\int_{0}^{t_{\text{now}}}dx_{c}(g,t)=\alpha_{c}\int_{0}^{t_{\text{now}}}dx(g,t)\forall g.$$

For short time intervals in which the changes in *n*_*c *_are small relative to *n*_*c *_itself, the changes in *x*_*c *_are related to the changes in *n*_*c *_through

(8) $$dx_{c}(g,t)=\frac{dn_{c}(g,t)}{n_{c}(g,t)},$$

and similarly

(9) $$dx(g,t)=\frac{dn(g,t)}{n(g,t)}.$$

Substituting these in (7) we obtain

(10) $$\alpha_{c}=\frac{\int_{0}^{t_{\text{now}}}\frac{dn_{c}(g,t)}{n_{c}(t)}}{\int_{0}^{t_{\text{now}}}\frac{dn(g,t)}{n(g,t)}}\forall g.$$

**Figure 2:**
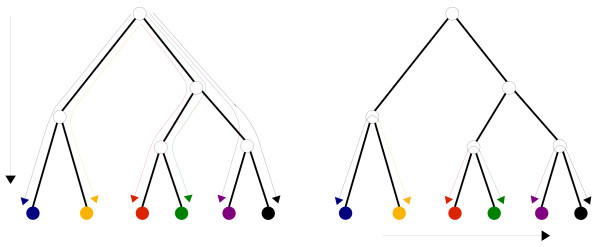
**Evolutionary histories and time invariance**. Evolutionary histories of different organisms. The scaling laws constrain integrals of domain-count changes over long evolutionary times, i.e. from the common ancestor up to the present (left panel). Our assumption of time invariance now implies relations between the domain-count changes during short time intervals which can be tested by comparing domain-counts in closely-related genomes (right panel).

Equation (10) summarizes the implications for domain-count dynamics implied by the scaling laws. It states that, *independent *of which evolutionary history we take, the ratio of the integrals of *dn*_*c*_/*n*_*c *_and *dn*/*n *over all evolutionary time must match the scaling exponent *α*_*c*_. This is illustrated on the left-hand side of figure 2, i.e. equation (10) implies that the ratio of integrals is the same for each of the evolutionary histories indicated as colored lines.

### Time Invariance

The equations (10) reflect the constraints on domain-count dynamics implied by the scaling laws but they don't uniquely determine an evolutionary model. To derive a unique evolutionary *null model *we will assume *time invariance *of the scaling laws. We assume that, if we had collected genomes of bacteria living several tens or even hundreds of million years ago, as opposed to the bacteria living today, we would have observed the *same *scaling laws as we observe today. That is, we assume that there is nothing particularly special about our current time, and that the same scaling laws have held since the last common ancestor, or at least since the origin of the clades from which our current genome sequences derive. We feel that this is by far the simplest assumption that can be made about the evolutionary dynamics and will here analyze its implications.

Given that the scaling laws are invariant in time, we immediately obtain that (10) should hold for each *short *time interval, i.e. we have that

(11) $$\frac{dn_{c}(g,t)}{n_{c}(g,t)}=\alpha_{c}\frac{dn(g,t)}{n(g,t)}\forall g,t,$$

or

(12) $$\frac{dn_{c}(g,t)}{dn(g,t)}=\alpha_{c}\frac{n_{c}(g,t)}{n(g,t)}\forall g,t.$$

That is, the assumption of time invariance implies that, for each genome *g*, and for each short time interval in its evolution, the ratio between the change *dn*_*c*_(*g*, *t*) in the domain-count of category *c *and the total change *dn*(*g*, *t*) in domain-count is given by the product of the exponent *α*_*c *_and the fraction *n*_*c*_(*g*, *t*)/*n*(*g*, *t*) of all domains that are of category *c*. In particular, equation (12) will apply to the domain-count changes that occurred since the common ancestors of pairs of closely-related species, as illustrated on the right-hand side of Fig. 2. Therefore, we can test the validity of the null model by comparing the domain-counts in the genomes of closely-related bacteria.

### Implications for closely-related pairs of genomes

We now discuss how the prediction (12) can be tested with data from closely-related genomes. Note that, strictly speaking, (12) holds only in the limit of infinitesimally small *dn*(*g*, *t*) and that we have so far implicitly assumed that the *n*_*c*_(*g*, *t*) are continuous variables, whereas in reality the smallest possible change is *dn*(*g*, *t*) = 1. For the integer-valued quantities *n*_*c*_(*g*, *t*) equation (12) can be interpreted as follows: whenever a single domain is added to the genome, i.e. *dn *= 1, then the *probability *that this domain is of category *c *is given by *α*_*c*_*n*_*c*_/*n*. Similarly, whenever a single domain is removed, i.e. *dn *= -1, then the probability that this domain is of category *c *is also given by *α*_*c*_*n*_*c*_/*n*.

Since this interpretation is of key conceptual importance we briefly expand on its meaning.

Mathematically, equation (10) makes a statement about the total overall changes in domain counts that happen over some finite time interval. In particular, the total change *dn*_*c *_that occurs over some time interval is the *difference *between the number of additions and deletions that occurred during that time interval. From a mathematical point of view, equation (11) is a differential equation that makes a statement about the relative *rates *at which changes in domain-count number occur, i.e. including both additions and deletions. To put it differently, the assumption of time invariance allows us to make statements about time intervals so short that at most one 'event' can occur during such intervals, so that there is roughly speaking no room left for additions and deletions to cancel each other out, i.e. the relation (11) must hold for both of them. The clearest interpretation is in terms of a model where the key quantities are the *rates*, i.e. probability of an event per unit time, at which domain-count changes (either additions or deletions) take place. That is, if *r *denotes the overall rate at which additions or deletions occur, and *r*_*c *_the rate at which additions/deletions of domains of category *c *occur, then the model predicts

(13) $$\frac{r_{c}}{r}=\alpha_{c}\frac{n_{c}}{n}.$$

For pairs of closely-related genomes the number of domain-count changes that occurred since they diverged from a common ancestor is generally very small compared to the total number of domains. Therefore, the fractions *n*_*c*_/*n *have generally changed little during the time since the two genomes diverged from their ancestor and we will make the assumption that the fraction *n*_*c*_/*n *can be considered constant. Under this approximation equation (13) predicts that, if during the time interval since the pair's common ancestor, a total of Δ*n *domain-count changes occurred, i.e. the *sum *of all additions and deletions, then the expected number of domain-count changes Δ*n*_*c *_in category *c *(which is again the *sum *of all additions and deletions in this category) should equal $\alpha_{c}\frac{n_{c}}{n}\Delta n$

We collected 93 pairs of fully-sequenced genomes that are evolutionary relatively closely related, using the tree of life that was inferred by Ciccarelli et al. [16] as a guide (Methods). For each pair of genomes *i *we counted the numbers of domain occurrences for each Pfam family and used these (Methods) to estimate the number of domain-count changes $\Delta n_{c}^{i}$ for each category *c *and the total number of domain-count changes Δ*n*^*i*^. Again, we stress that the $\Delta n_{c}^{i}$ are the estimated total number of changes, adding additions and deletions together. For example, if we denote by $dn_{c}^{i}$ the *difference *in the number of domains in category *c *occurring in the two genomes of the pair, then we typically find that the estimated $\Delta n_{c}^{i}$ is larger than $dn_{c}^{i}$ (see Additional file 1). Apart from estimating $\Delta n_{c}^{i}$ we estimated, for each genome pair *i*, the fractions $n_{c}^{i}/n^{i}$ by averaging the domain counts over the two genomes in the pair (Methods). Our model thus predicts that, for each pair *i*, the ratio $\Delta n_{c}^{i}/\Delta n^{i}$ should be proportional both to the fraction $n_{c}^{i}/n^{i}$ and to scaling law exponent *α*_*c*_.

### The fraction of domain-count changes is proportional to the number of existing domains

**Figure 3:**
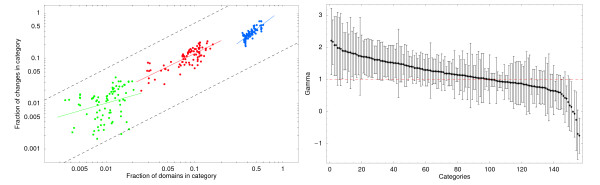
**Linear dependence of domain-count changes on domain occurrence**. Linear dependency of the fraction of domain-count changes on the domain-count itself. **Left panel **: For each genome pair *i *the fraction $\Delta n_{c}^{i}/\Delta n^{i}$ of domain-count changes that involve domains of category *c *is shown (vertical axis) as a function of the fraction $n_{c}^{i}/n^{i}$ of all domains in the genome that are associated with category *c *(horizontal axis) for the categories 'metabolic process' (blue), 'regulation of transcription' (red), and 'protein kinase activity' (green). Each dot corresponds to the data for one pair *i *of closely-related genomes. Both axes are shown on a logarithmic scale. The straight-lines show least-squares fits of the form $\log[\Delta n_{c}^{i}/\Delta n^{i}]=\gamma_{c}\log[n_{c}^{i}/n^{i}]+\delta_{c}$. The fitted slopes for the three categories are *γ*_prot.kin.activity _= 0.56 ± 0.46, *γ*_reg.transcr. _= 0.95 ± 0.20, and *a*_met.proc. _= 1.48 ± 0.31. For comparison the dotted lines show linear scaling. **Right panel**: A 99% posterior probability interval for the slope *γ*_*c *_was estimated for all selected GO categories (Methods). The fitted slopes were ordered from high to low and are shown in the right panel from left to right with the vertical bars corresponding to the 99% posterior probability intervals for each slope *γ*_*c*_. The slope *γ *= 1, corresponding to a linear dependency, is shown as a horizontal dotted line.

Equation (13) puts very strong constraints on the dynamics of domain-counts which we will check in three steps. First, we check that, for each category *c*, the estimated fractions Δ*n*_*c*_/Δ*n *of domain-count changes grow linearly with the fractions *n*_*c*_/*n*. The left panel of figure 3 shows scatter plots of $\Delta n_{c}^{i}/\Delta n^{i}$ as a function of $n_{c}^{i}/n^{i}$ for three selected categories. The axes are shown on logarithmic scales and the straight lines show least-squares linear fits of the form $\log[\Delta n_{c}^{i}/\Delta n^{i}]=\gamma_{c}\log[n_{c}^{i}/n^{i}]+\delta_{c}$.

The left panel of Fig. 3 demonstrates two points. First, comparing the three categories with each other, we see that most domain-count changes occur in the most abundant category and least domain-count changes occur in the least abundant category, with the fraction of domain-count changes $\Delta n_{c}^{i}/\Delta n^{i}$ indeed scaling roughly linearly with $n_{c}^{i}/n^{i}$ (compare with the dotted guide lines showing linear scaling). Beyond that, if we compare the numbers of domain-count changes across the different genomes *within *each category we see that, in those genomes where the domains of the category are most abundant domain-count changes in that category are also most abundant. That is, although the data is quite noisy, it is clear that all three clouds of points show a close to linear increase of $\Delta n_{c}^{i}/\Delta n^{i}$ with $n_{c}^{i}/n^{i}$.

The estimated slopes *γ*_*c *_for all selected GO categories are shown in the right panel of Fig. 3 (and listed in Additional file 1). The estimated *γ*_*c *_are very roughly symmetrically distributed around 1 with a median *γ*_*c *_of 1.16. For almost 75% of the categories a slope of *γ*_*c *_= 1 is within the 99% posterior probability interval.

This thus supports the prediction of our evolutionary null model that the fraction of all domain-count changes that involve domains of category *c *is proportional to the fraction *n*_*c*_/*n *of all domains in the genome that belong to category *c*.

For about 25% of the categories we infer slopes significantly deviating from 1. It should be noted, however, that the least-squares fitting assumes simple Gaussian noise in log[Δ*n*_*c*_/Δ*n*], whereas in reality the size of the noise in log[Δ*n*_*c*_/Δ*n*] increases as Δ*n *decreases. Moreover, whereas the fitting assumes that the numbers of domain-count changes are given, in reality these are estimated (see Methods) and thus themselves subject to uncertainty. We therefore are significantly underestimating the uncertainty in the fitted slope for many categories, and it is reasonable to conclude that for most if not all categories the data is consistent with the predicted linear dependence of Δ*n*_*c*_/Δ*n *on *n*_*c*_/*n*.

### Evolutionary Potentials

The results of the previous section support that the rate *r*_*c *_of domain-count changes involving domains of category *c *is proportional to the number of domains *n*_*c *_currently present in the genome. Let $r_{c}^{i}$ denote the rate of addition/deletion of domains of category *c *for genome pair *i *and let *r*^*i *^denote the overall rate of addition/deletion of domains for genome pair *i*. Assuming only that $r_{c}^{i}$ is proportional to $n_{c}^{i}$ we can generally write for the relative rates

(14) $$\frac{r_{c}^{i}}{r^{i}}=\rho_{c}^{i}\frac{n_{c}^{i}}{n^{i}},$$

which is a generalization of equation (13). The proportionality constants $\rho_{c}^{i}$ defined by this equation quantify the extent to which domain-count changes of category *c *are more or less frequent in the lineages of pair *i *than expected based on their frequency $n_{c}^{i}/n^{i}$. For this reason we will refer to these proportionality constants as *evolutionary potentials*. That is, when $\rho_{c}^{i}$ is high it indicates that, apparently, domain additions and deletions involving domains of category *c *are fixed in evolution at a higher rate in the evolutionary lineages of pair *i*.

Our evolutionary null model predicts that the evolutionary potentials $\rho_{c}^{i}$ are the same for all evolutionary lineages, and in addition that the evolutionary potentials $\rho_{c}^{i}$ are equal to the scaling law exponents *α*_*c*_. We will check these two predictions in turn.

### The evolutionary potentials $\rho_{c}^{i}$ are constant across evolutionary lineages

Given the estimated numbers of domain-count changes $\Delta n_{c}^{i}$, and the total number of domain-count changes Δ*n*^*i *^we can estimate the lineage-specific evolutionary potentials $\rho_{c}^{i}$ as follows. For every domain-count change that occurs, the probability that it will involve a domain of category *c *is simply given by the relative rate $r_{c}^{i}/r^{i}$. Therefore, if Δ*n*^*i *^domain-count changes occur in total, the probability that $\Delta n_{c}^{i}$ involve domains of category *c *is simply given by

(15) $$\begin{array}{r} P(\Delta n_{c}^{i}|\Delta n^{i},\rho_{c}^{i})=\left(\begin{array}{c} \Delta n^{i}\\ \Delta n_{c}^{i} \end{array}\right){\left(\rho_{c}^{i}\frac{n_{c}^{i}}{n^{i}}\right)}^{\Delta n_{c}^{i}}\\ {\left(1-\rho_{c}^{i}\frac{n_{c}^{i}}{n^{i}}\right)}^{\Delta n^{i}-\Delta n_{c}^{i}}, \end{array}$$

where we used the definition (14). Using a uniform prior over $\rho_{c}^{i}$ we and for the posterior probability of $\rho_{c}^{i}$ given the estimated domain-count changes

(16) $$\begin{array}{r} P(\rho_{c}^{i}|\Delta n^{i},\Delta n_{c}^{i})d\rho_{c}^{i}=\frac{n_{c}^{i}}{n^{i}}\frac{(\Delta n^{i}+1)!}{\Delta n_{c}^{i}!(\Delta n^{i}-\Delta n_{c}^{i})!}\\ {\left(\rho_{c}^{i}\frac{n_{c}^{i}}{n^{i}}\right)}^{\Delta n_{c}^{i}}{\left(1-\rho_{c}^{i}\frac{n_{c}^{i}}{n^{i}}\right)}^{\Delta n^{i}-\Delta n_{c}^{i}}d\rho_{c}^{i} \end{array}$$

**Figure 4:**
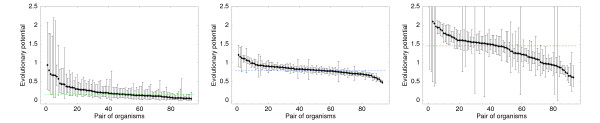
**Evolutionary potentials across different lineages**. Distribution of inferred evolutionary potentials $\rho_{c}^{i}$ for the categories 'translation' (left panel), 'metabolic process' (middle panel), and 'regulation of transcription' (right panel) across all genome pairs *i*. Each panel shows the 99% posterior probability intervals $\left[l_{c}^{i},h_{c}^{i}\right]$ for the potentials $\rho_{c}^{i}$ as vertical bars (sorted from left to right by their means). The dotted horizontal lines show the average $\rho_{c}^{i}$, averaged over all pairs *i*.

Using (16) we determined posterior probability intervals $\left[l_{c}^{i},h_{c}^{i}\right]$ defined by

(17) $$\int_{0}^{l_{c}^{i}}P(\rho |\Delta n^{i},\Delta n_{c}^{i})d\rho =0.01,$$

and

(18) $$\int_{0}^{h_{c}^{i}}P(\rho |\Delta n^{i},\Delta n_{c}^{i})d\rho =0.99,$$

for each category *c *and each genome pair *i*. Figure 4 shows these posterior probability intervals, for all genome pairs *i*, for the categories 'translation', 'metabolic process', and 'regulation of transcription'.

Since the total number of domain-count changes Δ*n*^*i *^is often small, it is not surprising that the posterior probability intervals are often rather wide. In spite of this, it can be clearly seen that, consistent with the scaling exponents *α*_*c*_, $\rho_{c}^{i}$ is largest for the category 'regulation of transcription', and smallest for the category 'translation'. Moreover, Fig. 4 shows that the data by and large support the prediction that the potentials $\rho_{c}^{i}$ are *the same *for all evolutionary lineages. That is, for each of the three categories the posterior probability intervals for $\rho_{c}^{i}$ are consistent with a common underlying potential *ρ*_*c *_for the majority of genome pairs *i*. This is a further piece of support for the evolutionary null model.

### Evolutionary potentials *ρ*_*c *_correlate with scaling exponents *α*_*c*_

The previous section has shown that the data are mostly consistent with constant evolutionary potentials across the genome pairs. We will now assume that the evolutionary potentials $\rho_{c}^{i}$ all equal a common potential *ρ*_*c *_and estimate it by combining data from all genome pairs. We find for the probability of *ρ*_*c *_given the observed domain-count changes {$\Delta n_{c}^{i}$} and {Δ*n*^*i*^}

(19) $$\begin{array}{r} P(\rho_{c}|\{\Delta n_{c}^{i}\},\{\Delta n^{i}\})\propto \prod_{i}{\left(\rho_{c}\frac{n_{c}^{i}}{n^{i}}\right)}^{\Delta n_{c}^{i}}\\ {\left(1-\rho_{c}\frac{n_{c}^{i}}{n^{i}}\right)}^{\Delta n^{i}-\Delta n_{c}^{i}}. \end{array}$$

**Figure 5:**
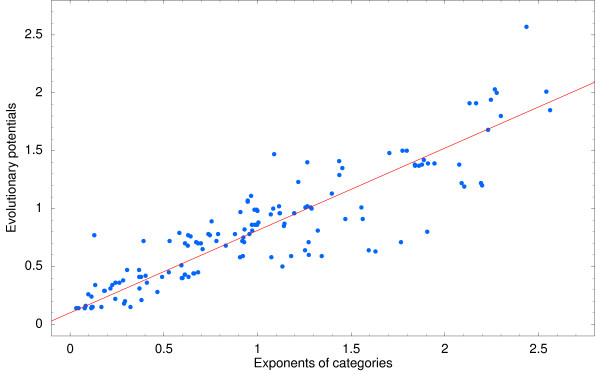
**Correlation between exponents *α*_*c *_and evolutionary potentials *ρ *_*c*_**. Correlation between the inferred evolutionary potentials *ρ*_*c *_(vertical axis) and the exponents *ρ*_*c *_(horizontal axis) of the scaling laws. Each dot corresponds to one of the 156 selected GO categories. The line shows the linear fit *ρ*_*c *_= 0.71*α*_*c *_+ 0.1 with correlation coefficient *r*^2 ^= 0.80.

Using this equation we estimated *ρ*_*c *_for each selected category *c*. Equation (13) predicts that the evolutionary potentials *ρ*_*c *_equal the scaling exponents *α*_*c*_. Figure 5 shows a scatter plot of *α*_*c *_against the estimated *ρ*_*c*_.

Note that, since the evolutionary potential *ρ*_*c *_is a measure of the relative frequency of domain-count changes between closely-related species, and *α*_*c *_is a measure of the scaling of the number of domains with genome size, there is *a priori *no reason why these two quantities should be strongly correlated. However, as predicted by our evolutionary null model, there is clear evidence of a linear dependency between the exponents *α*_*c *_and the evolutionary potentials *ρ*_*c*_.

Rather than a simple relation *ρ*_*c *_= *α*_*c *_we find that *ρ*_*c *_varies over a somewhat smaller range, i.e. the 99% posterior probability interval for the slope of the correlation runs from 0.65 to 0.79. One possible explanation is that, because the estimation of the numbers of domain-count changes Δ*n*_*c *_is the same for all categories, we might underestimate the numbers of domain-count changes more for categories with large *ρ*_*c *_than for categories with low *ρ*_*c*_.

### Implications for the rates of horizontal transfer

In general, the rate at which additions/deletions occur is the product of two independent factors. First, the rate at which domain additions and deletions are *introduced *into individuals of the population, and second the fraction of the time that such mutations are being fixed into the population. There are likely three main mechanisms through which domain additions or deletions are introduced: duplications, deletions, and horizontal transfers. To a first approximation, the rates at which duplications, deletions, and horizontal transfers are being introduced into individuals will be determined by the biases inherent in the mechanisms underlying these processes and not by selection. In contrast, the fraction of the time that such mutations are fixed in evolution will strongly depend on selection.

It is clear that, for duplications and deletions, the rate at which such mutations are introduced is naturally proportional to the number of existing domains *n*_*c*_. That is, when the number of domains *n*_*c *_doubles, the total rate at which duplications and deletions are introduced within this category also doubles. Moreover, since selection is not involved, the rate of introduction of duplications and deletions can be expected to be the *same *for all functional categories *c *(except of course for transposable elements which are duplicated through a separate mechanism). Therefore, as the rate of introduction is proportional to *n*_*c*_, with the same proportionality constant for each category, and the total rate must be proportional to *ρ*_*c*_*n*_*c*_, this implies that the relative rate of *fixation *through selection must be proportional to the evolutionary potential *ρ*_*c*_.

Thus, the evolutionary potentials *ρ*_*c *_(and the scaling exponents *α*_*c*_) have a particularly simple interpretation: they give the average relative rate with which additions and deletions of domains in category *c *are fixed by selection.

Evidence has accumulated over recent years that horizontal transfers occur in essentially all evolutionary lineages and gene families, see e.g. [17-22] and the extensive discussion in the recent review [23]. There is less consensus in the literature, however, regarding the precise amount that horizontal transfer contributes to gene-content evolution. Discussion of the reasons for this lack of consensus are beyond the scope of this article but it is clear that the apparent disparity between the conclusions reached by different authors is a combination of: the fact that different authors ask different questions, i.e. asking what fraction of gene families are affected by horizontal transfer at least once in evolutionary history [24] is very different from asking, for example, the relative rates of gene loss to horizontal transfer [25,26]. The fact that very different types of evidence are used, such as presence/absence of members of a gene family across leafs of the species tree [19,20,26], comparison of gene trees with species trees [21,27], or the presence of genes without known homologs [28]. And finally, the fact that there are technical issues (like the way gene families are build and tree topologies are inferred) which may affect the results quantitatively if not qualitatively.

To the present authors the evidence currently in the literature does suggest that horizontal gene transfer accounts for a non-negligible and maybe even a large fraction of changes in gene content, at least among closely-related genomes. For example, it was found in [20] that up to 20% of the gene-content of proteo-*γ *bacteria consists of genes that have no homology with any of the other genes among all currently sequenced proteo-*γ *bacteria, but that *do *have homology with genes found outside of the proteo-*γ *clade. It is hard to see how this statistic could result from any process other than a high rate of horizontal transfer. It is thus worthwhile to investigate the implications of our current findings under the assumption that many of the domain additions are due to horizontal transfer.

Although we have no direct evidence, it is attractive to assume that the probability that a domain addition will be fixed in the population does *not *depend on the mechanism by which it was introduced. That is, the relative rate of fixation of domain additions in category *c *should be proportional to *ρ*_*c *_for both duplicated domains as well as horizontally transfered domains. If this is indeed the case, it follows immediately from the fact that the overall rate should be proportional to *ρ*_*c*_*n*_*c*_, that the rate at which horizontal transfers are *introduced *must be proportional to the number of domains *n*_*c *_present in the genome. However, whereas this is naturally the case for gene duplications, it is not clear at all why this should also hold for horizontal transfers. Therefore, our results put rather strong constraints on the rate of horizontal transfer.

One possibility is that horizontal transfer is negligible and that domain additions are dominated by duplications. However, as we have just discussed, this assumption, which we have made in previous work [10,11], appears at odds with recent work. It should be noted, however, that some studies that investigate evolution of gene content over long time scales find that horizontal transfer is only responsible for a minor fraction of all events on a long time scale, i.e. [21]. One hypothesis that might be worthwhile to entertain is that most horizontal transfers are only transient. It is conceivable that horizontally transfered genes consist mostly of 'accessory' genes that are involved with adaptations to the local environment that are easily taken up by genomes moving into a certain environment, but which are also easily lost again when the environment changes, so that the horizontal transfers of these accessory genes contributes relatively little to the gene-content dynamics on long time scales. However, at least to these authors this hypothesis does not seem particularly plausible a priori.

Alternatively, there are several hypotheses that could explain why the rate at which horizontal transfers of domains of category *c *are introduced is proportional to the number of domains *n*_*c *_already in the genome. First, it is possible that horizontal transfer is highly biased to occur predominantly between genomes that are closely-related phylogenetically. One mechanism of horizontal gene transfer, conjugation, does indeed occur preferentially between related organisms. Since closely-related species are likely to have highly correlated domain counts, it is likely that the fraction *n*_*c*_/*n *of category *c *domains in the donor genome is close to the fraction of domains of category *c *in the receiver genome. However, many of the horizontal transfers detected through sequence analysis involve transfers between distally related species.

Another possible explanation is that bacterial habitats naturally separate into different genome-size classes. That is, it is conceivable that bacteria tend to be surrounded mostly by other bacteria of roughly the same genome size. Because the scaling laws apply to all genomes, the fractions *n*_*c*_/*n *are similar for similarly sized genomes and one would naturally have that the rate at which horizontal transfers of domains of category *c *occur is proportional to *n*_*c*_. As far as these authors are aware, currently there seems to be no evidence suggesting that there is a characteristic genome size for each bacterial habitat, but it appears that this hypothesis should in principle be testable using metagenomics data.

Finally, it is possible that, even though a given bacterium would generally be surrounded by other bacteria of very different sizes, that horizontal transfer is highly biased to occur predominantly between organisms that have genomes with similar sizes. In fact, there is some evidence in the literature that bacteria can recognize and silence horizontally transfered genes that have an AT-content which is significantly higher than the AT-content of the genome itself [29]. In addition, there is a good correlation between genome size and GC-content [30]. It is therefore conceivable that horizontal transfers between genomes of similar size are much more common than horizontal transfers between genomes of significantly different sizes.

In any case, whatever the underlying mechanism, if horizontal transfers account for a significant fraction of domain additions through evolution, then something must ensure that the rate of introduction of such horizontal transfers is proportional to the number of existing domains *n*_*c *_in the receiving genome.

## Conclusion

We have shown that, across all bacteria and for most high-level GO categories *c*, the number of domain occurrences *n*_*c *_scales as a power-law in the total number of domains *n*, with scaling exponents *α*_*c *_varying from close to zero to a bit larger than 2. We have derived what we believe is the simplest evolutionary model that can account for the observed scaling laws. This 'null model' assumes that, across all evolutionary lineages and all evolutionary times, the relative rate *r*_*c*_/*r *at which additions and deletions of domains of category *c *are fixed in evolution is proportional to the current fraction *n*_*c*_/*n *of domains in category *c *and a characteristic *evolutionary potential ρ*_*c *_which equals the scaling exponent *α*_*c*_.

By comparing genome-wide domain-counts *n*_*f *_for each Pfam family *f *across 93 pairs of closely-related species we have estimated the rates at which domain additions and deletions occur across GO categories and across different evolutionary lineages. The results of this analysis support the predictions made by the evolutionary null model. First, we have shown that, for most categories *c*, the relative rate *r*_*c*_/*r *of domain additions and deletions is proportional to the fraction of domains *n*_*c*_/*n *already occurring in the genome.

Second, we estimated the relative rates $r_{c}^{i}/r^{i}$ of domain additions and deletions independently for different evolutionary lineages *i *and used these to estimate lineage-dependent evolutionary potentials $\rho_{c}^{i}$. We found that, whereas the evolutionary potentials $\rho_{c}^{i}$ clearly vary between categories *c*, the data support the null model's prediction that for a given category *c *the potentials $\rho_{c}^{i}$ are the same across all evolutionary lineages *i*. Finally, by combining data from all lineages we estimated average evolutionary potentials *ρ*_*c *_and found that, as predicted by the model, there is a good correlation between these evolutionary potentials and the scaling law exponents *α*_*c*_. Importantly, this result establishes that there is a direct relation between the scaling of domain-counts with genome size and the rates with which domains are added and removed during short evolutionary time intervals. This reinforces our proposal that the evolutionary potentials *ρ*_*c *_are fundamental constants of the evolutionary process.

An interesting question is if our simple null model can also explain the observed power-law distribution [7-9] of genome-family sizes in each genome. In previous work [7] one of us has suggested that the simplest explanation for the power-law distribution of gene family sizes is a multiplicative noise process. Although we will defer a detailed analysis of the gene-family size distributions implied by our null model to future work, it is clear that the basic ingredients for such a multiplicative noise process are already present. Since the model only constrains the relative rates of domains in different functional categories, the overall rate of genome growth/shrinkage can fluctuate randomly, and the rates of different families within a functional category can also fluctuate around a common mean. It is interesting to note that our null model implies that categories with large exponents, such as transcription factors, should show larger fluctuations in gene family sizes than categories with small exponents. Since the categories with large exponents are more abundant in larger genomes this in turn implies that the exponent of the gene-family size distribution should increase (i.e. be less negative) for larger genomes. This is indeed what is observed [7].

If, as recent work suggests, horizontal transfer is an important force in shaping the gene-content of genomes, then our results put strong constraints on the rates *r*_*c *_at which horizontal transfers of domains of different functional categories *c *can occur. In particular, we find that the rate at which domains of category *c *are horizontally transfered into a genome must be proportional to the number of domains *n*_*c *_already existing in the receiving genome. An important avenue for future research is to clarify the underlying mechanism that is responsible for this surprising fact.

As our results have made plausible that the evolutionary potentials *ρ*_*c *_(and the corresponding scaling exponents *α*_*c*_) are fundamental constants of the evolutionary process that apply across all time and all evolutionary lineages, the major challenge is now to elucidate what determines these numbers. In this respect it is important to note that the functional categories *c *that we consider are taken directly from the human-defined Gene Ontology hierarchy and are thus rather subjective. A first challenge for future work is therefore to identify a procedure that divides domain families into functional groups in a more objective manner. Although difficult with the current amount of available data, one possible approach is to estimate evolutionary potentials *ρ*_*f *_for individual domain families and to investigate if these fall into a small number of natural classes. That is, it is conceivable that on some more fundamental level there are only a small number of distinct exponents, for example *α *= 0, *α *= 1, and *α *= 2, and that the observed scaling laws with more complex exponents are different mixtures of these more fundamental scaling laws. Finally, we believe that the exponents *α*_*c *_reflect fundamental design principles of bacterial life, maybe similar to the way geometry and architectural design principles demand that the number of windows in a building scales as the 2/3 power of the building's volume. Seen from this point of view the exponents *α*_*c *_encode crucial information about the basic design that is shared by all bacterial life.

## Methods

### Domain counts

We obtained all 630 currently available bacterial genomes from the NCBI database [31]. To count the number of occurrences of each Pfam domain in each fully sequenced bacterial genome we ran HMMer [32] using all Pfam models on all proteins encoded in each genome, as annotated in the NCBI reference file. We thus assume that there are no significant fluctuations in the quality of gene prediction across the genomes. A hit was considered a valid domain if its score was equal or bigger than the so-called *gathering score *of the model provided by the Pfam web site, and it did not overlap with any other hit of lower E-value. There were 4,732 Pfam domain families with at least one occurrence across the 630 bacterial genomes. To count the number of domain occurrences per functional category we used a mapping from Pfam domains to Gene Ontolology terms [15] which is available at . If a domain-family *f *maps to category *c *it will be associated with *c *and all parent categories of *c *in the Gene Ontology hierarchy.

### Bayesian fitting of exponents

We used a Bayesian model to fit a power-law of the form $n_{c}=e^{\beta_{c}}n^{\alpha_{c}}$ for each category *c*. We discard all genomes with zero counts, i.e. *n*_*c*_(*g*) = 0, for each category *c *and log-transform the remaining domain-counts, i.e. (*x*_*g*_, *y*_*g*_) = (log[*n*_*c*_(*g*)], log[*n*(*g*)]). We assume that the pairs (*x*_*g*_, *y*_*g*_) derive from a line *y*_*g *_= *αx*_*g *_+ *β *plus noise of unknown size in both *x*- and *y*-direction. In addition we assume a rotationally invariant prior for the slope *α*. Under these assumptions the posterior probability density for the slope *α *given the data *D *is given by

(20) $$P(\alpha |D)d\alpha \propto \frac{{\left(\alpha^{2}+1\right)}^{(G-3)/2}}{{\left(\sigma_{yy}+\sigma_{xx}\alpha^{2}-2\alpha \sigma_{xy}\right)}^{(G-1)/2}}d\alpha ,$$

where *G *is the number of genomes, *σ*_*xx *_is the variance of *x *values, *σ*_*yy *_the variance of *y *values, and *σ*_*xy *_the covariance of *x *and *y *values. Note that the optimal line in this procedure corresponds roughly, i.e. up to the effects of the rotationally-invariant prior, to the line that minimizes the sum of the squared orthogonal distances of the data points to the line. The latter also corresponds to the first principal component of the data.

We selected all GO categories that have nonzero count in at least 95% of the genomes (600 out of 630), where the fraction of the variance explained by the fit is at least 0.9375 (this corresponds to the average distance to the data-points from the fitted line being 0.25 or less of the average distance of the data-points to the center of mass of the scatter), and where the average number of domains (averaged over all genomes) is at least 5. This led to 156 categories listed in Additional file 1.

To estimate the exponents *γ*_*c *_we make use of the additional information that the noise in the fraction *f*_*c *_is almost certainly much smaller than the noise in *dn*_*c*_/*dn*. Therefore, to estimate *γ*_*c *_we use a model in which all noise is assumed to occur in the vertical component, i.e. as is done in standard regression. Using again a rotationally invariant prior the posterior density for the exponent *γ*_*c *_as a function of the data is given by

(21) $$P(\gamma |D)d\gamma \propto \frac{{\left(\gamma^{2}+1\right)}^{-3/2}}{{\left(\sigma_{yy}+\sigma_{xx}\gamma^{2}-2\gamma \sigma_{xy}\right)}^{(P-1)/2}}d\alpha ,$$

where *P *is the number of genome pairs, the *x*-values are now given by the log-fractions, i.e. $x_{i}=\log[f_{c}^{i}]$, and the *y*-values are the log-fractions of changes, i.e. $y_{g}=\log[\Delta n_{c}^{i}/\Delta n^{i}]$.

### Extracting closely-related pairs of bacteria

We extracted the phylogenetic tree of bacteria from the tree of life that was produced by Ciccarelli et al. [16] based on the concatenated protein sequences of 31 protein families. As shown previously [33], even strains that are so close that they traditionally would be considered the same species, i.e. more than 94% nucleotide identity between orthologous genes, can have substantial differences in their gene content. In selecting 'close' pairs of organisms we want, on the one hand, to be able to estimate relative rates, for which we need a large enough number of domain additions and deletions to have taken place. On the other hand, the further apart the organisms, the harder it is to accurately estimate the *total *number of addition and deletion events that have taken place (see below). We decided to select all pairs of species for which the average identity at the amino acid level of orthologous proteins was at least 0.75, i.e. distance less than 0.25. With this definition one of the most distant pairs considered was *Escherichia coli *and *Vibrio Cholerae*. To avoid redundancy and pairs with too few events, we clustered all genomes whose distances were 0.01 or less and took a single representative genome from each cluster. With these cutoffs we obtained 93 pairs of bacterial genomes which are listed in Additional file 1.

### Estimating domain-count changes Δ*n*_*c*_

We estimate the number of domain-count changes Δ*n *and Δ*n*_*c *_by comparing domain counts for each Pfam family separately. Let $n_{f}^{1}$ and $n_{f}^{2}$ denote the number of occurrences of domains from family *f *in the first and second genome of the pair. We will assume that, during the time from the common ancestor of the two genomes, the rates at which domains were added and deleted for each family *f *is an unknown constant. In principle there are 4 unknown rates for each domain family *f*: the rate $\lambda_{f}^{1}$ at which domains of family *f *are added to genome 1, the rate at $\lambda_{f}^{2}$ which domains of family *f *are added to genome 2, the rate $\mu_{f}^{1}$ at which domains of family *f *are removed from genome 1, and the rate $\mu_{f}^{2}$ at which domains of family *f *are removed from genome 2. Since we cannot distinguish between additions to genome 1 and removals from genome 2 (and similarly for removals from genome 1 and additions to genome 2) we define the following rate sums

(22) $$\lambda_{f}=\lambda_{f}^{1}+\mu_{f}^{2},$$

and

(23) $$\mu_{f}=\lambda_{f}^{2}+\mu_{f}^{1}.$$

We denote by *a*_*f *_the number of additions in genome 1 plus deletions in genome 2, and by *d*_*f *_the number of additions in genome 2 plus deletions in genome 1. Since the rates of additions and deletions are assumed constant during the time interval since the common ancestor of the two genomes, both *a*_*f *_and *d*_*f *_are Poisson distributed

(24) $$P(a_{f},d_{f}|\lambda_{f},\mu_{f},t)=\frac{{\left(\lambda_{f}t\right)}^{a_{f}}{\left(\mu_{f}t\right)}^{d_{f}}}{a_{f}!d_{f}!}e^{-(\lambda_{f}+\mu_{f})t}$$

The expected total number of additions is

(25) $$\lambda =\sum_{f}\lambda_{f}t,$$

and the expected total number of deletions is given by

(26) $$\mu =\sum_{f}\mu_{f}t.$$

Next, we assume that the relative rate of additions involving domains of family *f *is the same as the relative rate of deletions involving domains of family *f*, and we denote this relative rate by *x*_*f*_, i.e.

(27) $$x_{f}=\frac{\lambda_{f}t}{\lambda }=\frac{\mu_{f}t}{\mu }.$$

In terms of these variables the probability of obtaining the set of additions and deletions {*a*_*f*_, *d*_*f*_} is

(28) $$P(\{a_{f},d_{f}\}|\lambda ,\mu ,\{x_{f}\})=\prod_{f}\frac{{\left(\lambda x_{f}\right)}^{a_{f}}}{a_{f}!}\frac{{\left(\mu x_{f}\right)}^{d_{f}}}{d_{f}!}e^{-(\lambda +\mu )}.$$

Assume that the number $n_{f}^{1}$ of domains of family *f *in genome 1 is bigger than the number $n_{f}^{2}$ of domains of family *f *in genome 2 and denote by *δn*_*f *_the difference, i.e. $\delta n_{f}=n_{f}^{1}-n_{f}^{2}$. We know that the number of additions *a*_*f *_must be at least *δn*_*f*_. Let *e*_*f *_the number of "extra" additions. Note that the number of deletions *d*_*f *_is then necessarily equal to *e*_*f *_. Similarly, if $n_{f}^{2}$ > $n_{f}^{1}$ we define, $\delta n_{f}=n_{f}^{2}-n_{f}^{1}$ and we write *d*_*f *_= *δn*_*f *_+ *e*_*f*_, and *a*_*f *_= *e*_*f*_. In terms of the *δn*_*f *_and the extra moves *e*_*f *_the probability is given by

(29) $$\begin{array}{r} P(\{\delta n_{f},e_{f}\}|\lambda ,\mu ,\{x_{f}\})=e^{-(\lambda +\mu )}\lambda^{A+E}\mu^{D+E}\\ \prod_{f}\frac{{\left(x_{f}\right)}^{\delta n_{f}+2e_{f}}}{e_{f}!(\delta n_{f}+e_{f})!}, \end{array}$$

where we have defined

(30) $$A=\sum_{f|n_{f}^{1}>n_{f}^{2}}\delta n_{f},$$

(31) $$D=\sum_{f|n_{f}^{2}>n_{f}^{1}}\delta n_{f},$$

and

(32) $$E=\sum_{f}e_{f}.$$

To estimate the number of additions and deletions for each family *f *we maximize the probability (29) with respect to *λ*, *μ*, the fractions *x*_*f*_, and the number of extra moves *e*_*f*_. To do this we use an iterative procedure. Note that, given the numbers of extra moves *e*_*f*_, the optimal *λ*, *μ*, and *x*_*f *_are given by

(33) *λ *= *A *+ *E*,

(34) *μ *= *D *+ *E*,

and

(35) $$x_{f}=\frac{\delta n_{f}+e_{f}}{\sum_{\tilde{f}}\delta n_{\tilde{f}}+e_{\tilde{f}}}.$$

Similarly, when the *x*_*f *_are given, the probability of *e*_*f *_conditioned on these variables is given by

(36) $$P(e_{f}|\lambda ,\mu ,x_{f},\delta n_{f})\propto \frac{{\left(x_{f}\right)}^{\delta n_{f}+2e_{f}}}{e_{f}!(\delta n_{f}+e_{f})!},$$

and we can numerically solve for the *ef *that maximizes this likelihood. We start by setting all *e*_*f *_= 0 and use the above equations to, iteratively, solve for *λ*, *μ *and the *x*_*f *_given the *e*_*f*_, and then the *e*_*f *_given the *x*_*f *_. This is repeated until a fixed point is reached. Finally, the estimated total number of events Δ*n*_*f *_for family *f *equals *δ n*_*f *_+ 2*e*_*f *_. In this way we estimate the number of events $\Delta n_{f}^{i}$ separately for each of the genome pairs *i *we analyze.

We originally performed this procedure including all Pfam domains. However, doing this we found that the number of extra moves *e*_*f *_estimated for categories associated with transposons and bacteriophages was many times larger than for all other families. This is of course to be expected as both transposons and phages actively multiply their domains. However, in equations (33) and (34) all domain families are treated equally, and therefore the high rates of additions and deletions for transposon and phage related categories significantly increase the estimated total rates for all families. Therefore, recognizing that the mechanisms of domain additions in transposon and phage related families are different from all other domain families, we excluded those Pfams associated with transposons and bacteriophages. In particular, we excluded all 22 Pfam families that map to the GO categories transposition (GO:0032196) or viral reproduction (GO:0016032).

The estimated total number of changes in category *c *is given by $\Delta n_{c}^{i}=\sum_{f\in c}\Delta n_{f}^{i}$, where the sum is over all Pfam domain families *f *associated with category *c*. The estimated total number of changes is given by $\Delta n^{i}=\sum_{f}\Delta n_{f}^{i}$, where the sum is over all Pfam domain families. To calculate the fractions $n_{c}^{i}/n^{i}$ for a given closely-related pair *i *we calculate the average number of domains associated with category *c *as $n_{c}^{i}=\sum_{f\in c}(n_{f}^{1}+n_{f}^{2})/2$ and the average total number of domains $n^{i}=\sum_{f}(n_{f}^{1}+n_{f}^{2})/2$.

## Competing interests

The authors declare that they have no competing interests.

## Authors' contributions

EvN conceived and designed the study and contributed to the methods development. NM performed the research. EvN and NM jointly wrote the paper.

## Reviewers' comments

Eugene V. Koonin

This is an important and welcome development of Van Nimwegen's 2003 classic on the scaling laws for different functional categories of genes in prokaryotic genomes. That classic study established that different functional classes of genes all scale according to power laws but with different, function-specific exponents that are (at least, approximately) the same in all prokaryotic lineages and, supposedly, the same throughout the course of prokaryotic evolution. This paper underpins the scaling laws with the (apparently) simplest conceivable evolutionary model. Under this model, the dynamics of protein domains in each functional category depends on just two variables, the number (fraction) of domains of the given category that are already present in the genome and the intrinsic evolutionary potential of the category. It is shown that the observations on the actual counts of domains in genomes are well explained if the evolutionary potentials are category-specific but invariant across bacterial lineages. All of the above had to be done in order to obtain a concrete evolutionary mechanism yielding the observed scaling laws but the above results are not at all unexpected. As I see it, the interesting things start coming up when one starts considering mechanism of domain gain in specific terms. For the model to work it is necessary that the rate at which horizontally transferred genes are acquired by a prokaryotes is proportional to the number of domains of the given category that are already present in the genome. Why that would be the case remains unclear. I believe the possibility that horizontal gene transfer is negligible compared to duplication as the source of new genes (domains) can be dismissed with confidence. In all, likelihood, in prokaryotes, horizontal gene transfer is actually a more important source of new genes than duplication. It is hard to think of a way for the domain content in the recipient organism could directly affect the rate of horizontal transfer. So the explanation should be indirect, that is, should include a connection between the domain composition of the donor genome with that of the recipient. Molina and Van Nimwegen consider three possibilities, and to me, the one that horizontal gene transfer predominantly occurs between genomes with similar AT-content (horizontally transferred genes coming from organisms with substantially different AT-content being rapidly destroyed or silenced) is highly attractive considering the strong correlation between AT-content and genome size. These hypotheses are testable by comparative-genomic methods although the analysis will not be easy. Of course, in the face of the rather counter-intuitive finding that the rate of horizontal gene transfer should depend on the fraction of domains of the given category already present, one has to consider the possibility that the proposed evolutionary model is too simple to be true. As far as I can see, more specifically, that would imply that the evolutionary potentials are not time-invariant and/or lineage-independent. The results of the present paper do not seem to point in this direction but I suspect that this is not the last word on the subject, more detailed analyses are necessary. On the whole, this is an enormously interesting subject, and the present paper is a useful stepping stone toward understanding the scaling laws. I am particularly intrigued by the final proposition that there could be only three fundamental exponents, the intermediate values currently observed depending on mixing of genes from the three classes in different proportions. Philosophically, this seems to smack of essentialism but... should there be a mechanistic explanation(s) of the 0, 1, and 2 scaling (and I can think of some), this would be a real step ahead in our understanding of how genomes evolve.

Martijn A. Huynen

The manuscript by Molina and van Nimwegen is the culmination of an observation that was originally made by Erik van Nimwegen and on which there has been follow op from several corners: that the variation in the number of proteins in a specific functional class across species scales as a power-law with the total number of proteins encoded in the species, and that the exponent of that power-law varies between the various classes.

Molina and van Nimwegen analyse their model further to show that the number of additions and losses within each category is proportional to the number of genes of that category already in the genome. They show that this prediction is borne out by comparing the number of genes in closely related genomes. Questions: In work of this referee and van Nimwegen we showed that the frequency distribution of gene family sizes in complete genomes follows a power-law, and we argued that this was only possible under a model in which the variations in the number of genes per gene family was proportional to the gene family size. Do I understand correctly that the model and observations in the Molina and van Nimwegen manuscript are consistent with this model, and more importantly, that these two observations about gene family size distributions 1) the size distribution of one family over genomes and 2) the size distribution of all families within one genome, can now be explained by one single model?

The authors argue for domains as the evolutionary unit. This may well be, but such a lower resolution does run the risk of mixing functional categories for domains that function in multiple categories. How many domains did map to multiple categories? And how did that affect the results?

With respect to Horizontal gene transfer the authors do not analyze this process per se, but rather argue that if it is frequent it should also be proportional to gene family size. I do not want to get into a whole HGT debate here, but, although over the complete history of life, along all evolutionary branches, few gene families appear to escape HGT, or at least escape evidence for HGT, compared to processes like gene duplication and gene loss, the quantitative contribution from any generation to the next appears to be small (Snel Bork and Huynen Genome Res 2002). There are other references (BG Mirkin, TI Fenner, MY Galperin, EV Koonin 2004) that do give higher estimates however. In any case it would be worthwhile to mention that relative to gene duplication and gene loss the amount of HGT that actually happens is not necessarily as large as is sometimes implicitly suggested.

With respect to the HGT mainly occurring between closely related species: there is evidence for that (e.g. by conjugation).

With respect to the closely related genomes: can the authors check whether the protein prediction in a pair of genomes was done with the same programs? or did they run the HMMs directly against the DNA? This is a bit nitpicking I know, but comparisons of closely related species have been confounded by inconsistent genome annotations in the past.

Sergei Maslov

The manuscript presents an interesting study of evolutionary implications of previously reported scaling laws in the functional content of bacterial genomes. While it does not answer the ultimate question of why this scaling exists in the first place, it methodically explores all its logical consequences reflected in genomes' evolutionary history.

An important result of this study is that the overall rate of gene (or domain as used in this manuscript) additions AND deletions scales linearly with the number of genes (domains) in a given functional category. This statement is in principle separate and independent from the scaling law itself since it counts the SUM of the rates of domain additions and deletions and not the DIFFERENCE between them.

Unfortunately, when this quantity (Δ*n*_*c *_proportional to [rate of additions+rate of deletions]*c*) is first introduced on page 7, readers could easily confuse it with just the net change in *n*_*c *_(denoted *dnc *and proportional to [rate of additions-rate of deletions]_*c*_). As a result they would miss one of the central points of the manuscript. I recommend that authors spend some extra time upfront explaining the differences between Δ*n*_*c *_and *dn*_*c *_and emphasizing that, a priory, these two quantities are not at all close to each other. To quantify this difference authors might quote the average value of – [rate of additions-rate of deletions]_*c*_–/[rate of additions+rate of deletions]_*c *_for their 93 pairs of genomes.

My other comment concerns the proposed "superuniversality" of evolutionary potentials (*ρ*_*c*_) of a individual functional categories. In general this study indicates that *ρ*_*c *_remains nearly the same for all species and at **all timescales **of evolution. I have previously observed (S. Maslov, unpublished) that in a group of VERY CLOSELY related genomes (28 fully sequences E. coli and Salmonella strains) the number of transcription factors violates the *N*^2 ^scaling in spite of a considerable range of genome sizes (from 4300 to 5800 genes). The best fit to the scaling exponent gives 0.3 instead of 2. This might indicate that evolutionary dynamics might in fact be rather different on very short timescale. This does not contradict the results of this study since (as explained in the Methods) authors have grouped together all very closely related species (AA substitution rate below 1%). However, I believe this observation deserves future scrutiny since it may shed an additional light on elementary evolutionary steps shaping functional contents of bacterial genomes.

Finally, I would like to offer another possibility of how the results of this study could be reconciled with the evidence of widespread Horizontal Gene Transfer (HGT) among bacteria (see section 2.9). One way to explain the linear correlation between the rate of fixed horizontal gene transfers and the number of genes in host's genome, is to assume that a SUCCESSFUL group of HGT-acquired genes needs to be functionally integrated with the rest of the genome. An example would be a HGT-transferred metabolic pathway that in order to contribute to the biomass production needs to be connected with the rest of the metabolic network of its host. Genomes with larger number of genes *n *have more places where a HGT-transferred pathway could be successfully connected and hence would be characterized by a proportionally larger Δ*n*_*HGT *_. In fact, my collaborators and I (S. Maslov, S. Krishna, K. Sneppen (2008) under review) have recently proposed a model of such pathway-by-pathway evolution to explain the quadratic scaling of the number of transcription factors with genome size.

## Supplementary Material

Additional file 1 Supplementary Materials. Additiona file 1 lists all 630 genomes used, all 93 closely-related genome pairs, and the estimated *α*_*c*_, *γ*_*c *_and *ρ*_*c *_for all 156 selected GO categories. It also shows a histogram of the ratio $r_{c}^{i}=dn_{c}^{i}/\Delta n_{c}^{i}$ between the total change in domain count $dn_{c}^{i}$ and the estimated total number of domain-count changes $\Delta n_{c}^{i}$. Click here for file
