# Supplementary material for: The evolution of domain-content in bacterial genomes
Source: Biol Direct. 2008 Dec 11;3:51. doi: 10.1186/1745-6150-3-51 (PMC2615428; doi:10.1186/1745-6150-3-51)
Supplement: Additional file 1 — Supplementary Materials. Additiona file 1 lists all 630 genomes used, all 93 closely-related genome pairs, and the estimated αc, γc and ρc for all 156 selected GO categories. It also shows a histogram of the ratio rci=dnci/Δnci between the total change in domain count dnci and the estimated total number of domain-count changes Δnci. [file 1745-6150-3-51-S1.pdf]

Supplementary Material to: The evolution of domain content in bacterial genomes

Nacho Molina and Erik van Nimwegen
Biozentrum, the University of Basel, and Swiss Institute of Bioinformatics
Klingelbergstrasse 50/70, 4056-CH, Basel, Switzerland,
email: j.molina@unibas.ch and erik.vannimwegen@unibas.ch

December 11, 2008

Inferred exponents and potentials for all selected categories

GO term	α_c	γ_c	ρ_c
ribosome	0.03 ± 0.01	1.5 ± 0.5	0.14 ± 0.03
structural constituent of ribosome	0.03 ± 0.01	1.5 ± 0.5	0.14 ± 0.03
ribonucleoprotein complex	0.04 ± 0.01	1.5 ± 0.6	0.14 ± 0.03
intracellular non membrane bounded organelle	0.08 ± 0.01	1.6 ± 0.4	0.14 ± 0.02
non membrane bounded organelle	0.08 ± 0.01	1.6 ± 0.4	0.14 ± 0.02
translation	0.08 ± 0.01	1.4 ± 0.4	0.16 ± 0.01
cytoplasmic part	0.10 ± 0.02	1.4 ± 0.5	0.26 ± 0.03
aminoacyl tRNA ligase activity	0.11 ± 0.01	1.5 ± 0.5	0.14 ± 0.03
ribonucleoprotein complex biogenesis and assembly	0.11 ± 0.02	1.1 ± 0.7	0.24 ± 0.09
tRNA aminoacylation for protein translation	0.12 ± 0.01	1.7 ± 0.6	0.15 ± 0.03
ligase activity forming aminoacyl tRNA and related compounds	0.12 ± 0.01	1.5 ± 0.6	0.15 ± 0.02
ligase activity forming carbon oxygen bonds	0.12 ± 0.01	1.5 ± 0.6	0.15 ± 0.02
amino acid activation	0.12 ± 0.01	1.6 ± 0.6	0.15 ± 0.02
tRNA aminoacylation	0.12 ± 0.01	1.6 ± 0.6	0.15 ± 0.02
RNA polymerase activity	0.13 ± 0.02	2.1 ± 0.9	0.77 ± 0.15
DNA directed RNA polymerase activity	0.13 ± 0.02	2.1 ± 0.9	0.77 ± 0.15
structural molecule activity	0.13 ± 0.02	1.1 ± 0.6	0.34 ± 0.03
tRNA metabolic process	0.17 ± 0.02	1.7 ± 0.6	0.15 ± 0.02
intracellular organelle	0.18 ± 0.02	1.6 ± 0.5	0.29 ± 0.02
organelle	0.18 ± 0.02	1.6 ± 0.5	0.29 ± 0.02
translation factor activity nucleic acid binding	0.21 ± 0.03	1.8 ± 0.7	0.31 ± 0.06
translation regulator activity	0.23 ± 0.03	1.8 ± 0.7	0.34 ± 0.07
gene expression	0.24 ± 0.01	1.4 ± 0.4	0.22 ± 0.01
macromolecular complex	0.24 ± 0.02	1.5 ± 0.4	0.36 ± 0.02
cytoplasm	0.26 ± 0.02	1.0 ± 0.3	0.36 ± 0.02
macromolecule biosynthetic process	0.28 ± 0.02	1.0 ± 0.3	0.38 ± 0.01
guanyl nucleotide binding	0.29 ± 0.02	0.9 ± 0.7	0.18 ± 0.02

guanyl ribonucleotide binding	0.29 ± 0.02	0.9 ± 0.7	0.18 ± 0.02
GTP binding	0.29 ± 0.02	0.9 ± 0.7	0.18 ± 0.02
RNA binding	0.29 ± 0.02	1.7 ± 0.5	0.20 ± 0.02
intracellular part	0.30 ± 0.02	1.3 ± 0.4	0.47 ± 0.01
RNA processing	0.32 ± 0.03	2.2 ± 0.7	0.15 ± 0.03
nucleotidyltransferase activity	0.37 ± 0.03	1.7 ± 0.6	0.47 ± 0.04
cellular protein metabolic process	0.37 ± 0.02	1.0 ± 0.3	0.41 ± 0.01
ligase activity	0.37 ± 0.02	1.3 ± 0.3	0.31 ± 0.02
protein metabolic process	0.38 ± 0.02	0.9 ± 0.3	0.41 ± 0.01
GTPase activity	0.38 ± 0.03	0.4 ± 0.8	0.21 ± 0.06
cellular macromolecule metabolic process	0.39 ± 0.02	1.1 ± 0.3	0.72 ± 0.01
cellular biosynthetic process	0.40 ± 0.01	1.4 ± 0.3	0.42 ± 0.01
nuclease activity	0.41 ± 0.03	1.5 ± 0.4	0.36 ± 0.03
RNA metabolic process	0.47 ± 0.03	1.8 ± 0.6	0.28 ± 0.02
isomerase activity	0.49 ± 0.03	1.7 ± 0.5	0.41 ± 0.03
biosynthetic process	0.53 ± 0.02	1.4 ± 0.3	0.45 ± 0.01
macromolecule metabolic process	0.53 ± 0.02	1.1 ± 0.3	0.72 ± 0.01
nucleobase nucleoside nucleotide and nucleic acid metabolic process	0.58 ± 0.03	1.4 ± 0.5	0.79 ± 0.01
nucleotide metabolic process	0.59 ± 0.03	1.9 ± 0.6	0.51 ± 0.04
nucleoside phosphate metabolic process	0.59 ± 0.03	1.9 ± 0.6	0.51 ± 0.04
amino acid metabolic process	0.60 ± 0.03	1.7 ± 0.6	0.40 ± 0.02
amino acid and derivative metabolic process	0.60 ± 0.03	1.7 ± 0.6	0.40 ± 0.02
nucleobase nucleoside and nucleotide metabolic process	0.61 ± 0.04	1.5 ± 0.6	0.43 ± 0.03
nucleobase nucleoside and nucleotide biosynthetic process	0.61 ± 0.03	0.9 ± 0.8	0.43 ± 0.08
primary metabolic process	0.61 ± 0.02	1.2 ± 0.3	0.70 ± 0.01
cellular metabolic process	0.63 ± 0.02	1.4 ± 0.3	0.68 ± 0.01
intracellular	0.63 ± 0.02	0.5 ± 0.6	0.77 ± 0.01
amine metabolic process	0.63 ± 0.03	1.6 ± 0.6	0.41 ± 0.02
biopolymer metabolic process	0.64 ± 0.04	1.2 ± 0.4	0.76 ± 0.01
carboxylic acid metabolic process	0.66 ± 0.03	1.6 ± 0.6	0.44 ± 0.02
organic acid metabolic process	0.66 ± 0.03	1.6 ± 0.6	0.44 ± 0.01
ribonucleotide binding	0.67 ± 0.02	0.8 ± 0.5	0.71 ± 0.01
purine ribonucleotide binding	0.67 ± 0.02	0.8 ± 0.5	0.71 ± 0.01
nitrogen compound metabolic process	0.68 ± 0.03	1.4 ± 0.6	0.45 ± 0.02
purine nucleotide binding	0.68 ± 0.02	0.8 ± 0.5	0.70 ± 0.01
nucleotide binding	0.70 ± 0.02	0.8 ± 0.5	0.70 ± 0.01
hydrolase activity acting on ester bonds	0.71 ± 0.03	0.9 ± 0.5	0.65 ± 0.03
adenyl ribonucleotide binding	0.74 ± 0.02	0.9 ± 0.6	0.78 ± 0.01
ATP binding	0.74 ± 0.02	0.9 ± 0.6	0.78 ± 0.01
adenyl nucleotide binding	0.75 ± 0.02	0.9 ± 0.6	0.77 ± 0.01
cellular process	0.76 ± 0.02	1.2 ± 0.5	0.89 ± 0.01
transferase activity transferring phosphorus containing groups	0.78 ± 0.03	1.5 ± 0.7	0.72 ± 0.03
metabolic process	0.79 ± 0.01	1.5 ± 0.3	0.78 ± 0.01
response to stress	0.83 ± 0.04	1.7 ± 0.7	0.68 ± 0.04
transferase activity	0.88 ± 0.02	1.8 ± 1.1	0.78 ± 0.01
transferase activity transferring one carbon groups	0.91 ± 0.04	0.7 ± 0.9	0.58 ± 0.04
binding	0.91 ± 0.01	0.8 ± 0.7	0.97 ± 0.01
peptidase activity	0.92 ± 0.05	1.3 ± 0.5	0.72 ± 0.04
methyltransferase activity	0.92 ± 0.04	1.1 ± 1.0	0.59 ± 0.04
cellular catabolic process	0.93 ± 0.04	1.1 ± 0.8	0.75 ± 0.05
proteolysis	0.93 ± 0.05	1.3 ± 0.5	0.71 ± 0.04
hydrolase activity	0.93 ± 0.03	1.2 ± 0.6	0.82 ± 0.01
cell part	0.95 ± 0.03	1.3 ± 0.5	1.06 ± 0.01
cellular component	0.95 ± 0.03	1.3 ± 0.5	1.07 ± 0.01
cell	0.95 ± 0.03	1.3 ± 0.5	1.06 ± 0.01

catabolic process	0.96 ± 0.04	0.8 ± 0.9	0.78 ± 0.04
nucleic acid binding	0.97 ± 0.03	1.5 ± 1.1	1.11 ± 0.01
pyrophosphatase activity	0.97 ± 0.05	1.0 ± 0.8	0.86 ± 0.03
hydrolase activity acting on acid anhydrides in phosphorus containing anhydrides	0.97 ± 0.05	1.0 ± 0.8	0.86 ± 0.03
catalytic activity	0.97 ± 0.01	1.9 ± 0.6	0.81 ± 0.01
biological process	0.98 ± 0.01	0.7 ± 1.0	0.99 ± 0.01
nucleoside triphosphatase activity	0.99 ± 0.05	1.0 ± 0.8	0.86 ± 0.03
molecular function	1.00 ± 0.00	1.3 ± 0.9	0.99 ± 0.00
hydrolase activity acting on acid anhydrides	1.00 ± 0.05	1.1 ± 0.7	0.86 ± 0.02
known function	1.00 ± 0.00	0.0 ± 0.0	0.98 ± 0.00
membrane part	1.00 ± 0.05	1.9 ± 0.9	0.88 ± 0.03
transition metal ion binding	1.07 ± 0.04	2.2 ± 1.0	0.95 ± 0.04
monocarboxylic acid metabolic process	1.07 ± 0.06	1.9 ± 0.9	0.58 ± 0.06
ATPase activity	1.08 ± 0.06	1.1 ± 0.7	1.00 ± 0.03
response to stimulus	1.09 ± 0.04	2.0 ± 1.0	1.47 ± 0.04
cation binding	1.11 ± 0.04	2.0 ± 1.1	1.02 ± 0.04
ion binding	1.12 ± 0.04	1.7 ± 1.2	0.96 ± 0.03
metal ion binding	1.12 ± 0.04	2.0 ± 1.1	0.96 ± 0.03
coenzyme metabolic process	1.13 ± 0.06	0.7 ± 0.8	0.50 ± 0.04
lyase activity	1.14 ± 0.05	0.6 ± 0.8	0.85 ± 0.04
kinase activity	1.14 ± 0.05	0.8 ± 1.0	0.87 ± 0.04
FAD binding	1.18 ± 0.06	1.8 ± 0.8	0.59 ± 0.07
integral to membrane	1.20 ± 0.05	1.1 ± 0.5	0.96 ± 0.04
intrinsic to membrane	1.20 ± 0.05	1.1 ± 0.5	0.96 ± 0.04
membrane	1.22 ± 0.03	1.4 ± 0.5	1.23 ± 0.01
oxidoreductase activity acting on CH OH group of donors	1.25 ± 0.06	0.1 ± 0.8	0.64 ± 0.04
establishment of localization	1.26 ± 0.05	1.2 ± 0.4	1.01 ± 0.01
localization	1.27 ± 0.05	1.2 ± 0.3	1.02 ± 0.01
DNA binding	1.27 ± 0.04	1.3 ± 0.5	1.40 ± 0.02
coenzyme binding	1.27 ± 0.04	0.3 ± 0.8	0.71 ± 0.04
oxidoreductase activity acting on the CH OH group of donors NAD or NADP as acceptor	1.27 ± 0.06	-0.3 ± 0.9	0.60 ± 0.04
transport	1.28 ± 0.06	1.2 ± 0.4	1.01 ± 0.01
transporter activity	1.29 ± 0.06	1.3 ± 0.4	1.00 ± 0.01
hydrolase activity acting on carbon nitrogen but not peptide bonds	1.32 ± 0.06	-0.1 ± 0.7	0.81 ± 0.06
aromatic compound metabolic process	1.34 ± 0.07	0.8 ± 0.8	0.59 ± 0.04
cofactor binding	1.40 ± 0.04	0.8 ± 0.6	1.13 ± 0.03
acyltransferase activity	1.44 ± 0.07	1.2 ± 0.7	1.41 ± 0.06
transferase activity transferring acyl groups	1.44 ± 0.06	1.0 ± 0.7	1.29 ± 0.05
transferase activity transferring groups other than amino acyl groups	1.45 ± 0.07	1.1 ± 0.8	1.35 ± 0.06
phosphoric ester hydrolase activity	1.47 ± 0.08	0.6 ± 0.5	0.91 ± 0.10
phosphotransferase activity alcohol group as acceptor	1.55 ± 0.08	1.0 ± 0.6	1.01 ± 0.04
oxidoreductase activity	1.56 ± 0.06	1.2 ± 0.5	0.91 ± 0.02
pyridoxal phosphate binding	1.59 ± 0.08	-0.7 ± 0.8	0.64 ± 0.05
transferase activity transferring nitrogenous groups	1.63 ± 0.09	0.2 ± 0.8	0.63 ± 0.07
biological regulation	1.70 ± 0.03	1.2 ± 0.2	1.48 ± 0.01
vitamin binding	1.77 ± 0.08	-0.7 ± 0.5	0.71 ± 0.04
regulation of biological process	1.77 ± 0.03	1.2 ± 0.2	1.50 ± 0.01
regulation of cellular process	1.80 ± 0.04	1.1 ± 0.2	1.50 ± 0.02
regulation of gene expression	1.84 ± 0.04	0.9 ± 0.2	1.38 ± 0.02
regulation of metabolic process	1.84 ± 0.04	1.0 ± 0.2	1.37 ± 0.03
regulation of cellular metabolic process	1.86 ± 0.04	1.0 ± 0.2	1.37 ± 0.03
regulation of nucleobase nucleoside nucleotide and nucleic acid metabolic process	1.88 ± 0.04	1.0 ± 0.2	1.38 ± 0.03

transcription regulator activity	1.89 ± 0.04	1.0 ± 0.2	1.42 ± 0.03
FMN binding	1.91 ± 0.12	0.4 ± 0.4	0.80 ± 0.10
regulation of transcription	1.91 ± 0.04	0.9 ± 0.2	1.39 ± 0.03
regulation of transcription DNA dependent	1.95 ± 0.04	0.9 ± 0.2	1.39 ± 0.03
regulation of RNA metabolic process	1.95 ± 0.04	0.9 ± 0.2	1.39 ± 0.03
transcription factor activity	2.08 ± 0.06	0.8 ± 0.2	1.38 ± 0.03
protein kinase activity	2.09 ± 0.11	0.6 ± 0.5	1.22 ± 0.07
phosphotransferase activity nitrogenous group as acceptor	2.10 ± 0.10	0.8 ± 0.5	1.19 ± 0.07
cell communication	2.13 ± 0.11	1.1 ± 0.4	1.91 ± 0.04
signal transduction	2.17 ± 0.11	1.1 ± 0.4	1.91 ± 0.04
two component sensor activity	2.19 ± 0.12	0.7 ± 0.5	1.22 ± 0.07
protein histidine kinase activity	2.19 ± 0.12	0.7 ± 0.5	1.22 ± 0.07
electron carrier activity	2.20 ± 0.16	0.8 ± 0.2	1.20 ± 0.04
two component signal transduction system phosphorelay	2.23 ± 0.10	0.9 ± 0.5	1.68 ± 0.06
acetyltransferase activity	2.25 ± 0.14	0.9 ± 0.6	1.94 ± 0.09
N acetyltransferase activity	2.27 ± 0.15	0.6 ± 0.5	2.03 ± 0.10
N acyltransferase activity	2.28 ± 0.14	0.6 ± 0.5	2.00 ± 0.10
two component response regulator activity	2.30 ± 0.12	0.9 ± 0.6	1.80 ± 0.07
response to chemical stimulus	2.44 ± 0.16	0.6 ± 0.3	2.57 ± 0.09
molecular transducer activity	2.54 ± 0.13	1.3 ± 0.4	2.01 ± 0.04
signal transducer activity	2.54 ± 0.13	1.3 ± 0.4	2.01 ± 0.04
sequence specific DNA binding	2.56 ± 0.12	0.8 ± 0.5	1.85 ± 0.06

List of genome pairs

Genome 1	Genome 2	Distance
Bacillus anthracis Ames	Bacillus cereus ATCC14579	0.012
Helicobacter pylori 26695	Helicobacter pylori J99	0.015
Salmonella enterica Choleraesuis	Escherichia coli 536	0.017
Xanthomonas campestris	Xanthomonas citri	0.020
Xylella fastidiosa M12	Xylella fastidiosa	0.021
Vibrio vulnificus YJ016	Vibrio parahaemolyticus	0.032
Streptococcus agalactiae 2603	Streptococcus pyogenes MGAS315	0.042
Chlamydia muridarum	Chlamydia trachomatis	0.046
Streptomyces coelicolor	Streptomyces avermitilis	0.054
Vibrio cholerae	Vibrio vulnificus YJ016	0.058
Staphylococcus aureus N315	Staphylococcus epidermidis RP62A	0.058
Pseudomonas putida F1	Pseudomonas syringae	0.059
Corynebacterium efficiens YS-314	Corynebacterium glutamicum	0.060
Vibrio cholerae	Vibrio parahaemolyticus	0.065
Mycobacterium avium paratuberculosis	Mycobacterium bovis	0.067
Rickettsia conorii	Rickettsia prowazekii	0.067
Pasteurella multocida	Haemophilus influenzae	0.072
Streptococcus agalactiae 2603	Streptococcus mutans	0.074
Streptococcus pyogenes MGAS315	Streptococcus mutans	0.081
Mycobacterium bovis	Mycobacterium leprae	0.082
Yersinia pestis biovar Mediaevails	Photorhabdus luminescens	0.085
Escherichia coli 536	Yersinia pestis biovar Mediaevails	0.085
Salmonella enterica Choleraesuis	Yersinia pestis biovar Mediaevails	0.086
Mycobacterium avium paratuberculosis	Mycobacterium leprae	0.087
Streptococcus pneumoniae R6	Streptococcus agalactiae 2603	0.088
Pseudomonas aeruginosa	Pseudomonas putida F1	0.088

Pseudomonas aeruginosa	Pseudomonas syringae	0.093
Streptococcus pneumoniae R6	Streptococcus pyogenes MGAS315	0.094
Streptococcus pneumoniae R6	Streptococcus mutans	0.100
Escherichia coli 536	Photorhabdus luminescens	0.102
Salmonella enterica Choleraesuis	Photorhabdus luminescens	0.102
Pasteurella multocida	Haemophilus ducreyi 35000HP	0.103
Rhodopseudomonas palustris BisA53	Bradyrhizobium japonicum	0.105
Haemophilus influenzae	Haemophilus ducreyi 35000HP	0.107
Rhizobium leguminosarum bv viciae 3841	Agrobacterium tumefaciens C58 UWash	0.108
Photobacterium profundum SS9	Vibrio vulnificus YJ016	0.116
Photobacterium profundum SS9	Vibrio cholerae	0.118
Prochlorococcus marinus MIT9313	Synechococcus sp WH8102	0.122
Photobacterium profundum SS9	Vibrio parahaemolyticus	0.123
Corynebacterium diphtheriae	Corynebacterium efficiens YS-314	0.127
Corynebacterium diphtheriae	Corynebacterium glutamicum	0.136
Chlamydophila caviae	Chlamydophila pneumoniae J138	0.164
Xanthomonas campestris	Xylella fastidiosa M12	0.170
Xanthomonas citri	Xylella fastidiosa M12	0.171
Bacillus subtilis	Bacillus anthracis Ames	0.171
Xanthomonas campestris	Xylella fastidiosa	0.175
Xanthomonas citri	Xylella fastidiosa	0.176
Bacillus subtilis	Bacillus cereus ATCC14579	0.177
Brucella melitensis	Rhizobium etli CFN 42	0.182
Chlamydia muridarum	Chlamydophila caviae	0.188
Chlamydia trachomatis	Chlamydophila caviae	0.195
Buchnera aphidicola	Buchnera aphidicola Sg	0.198
Lactococcus lactis	Streptococcus pneumoniae R6	0.199
Prochlorococcus marinus MED4	Prochlorococcus marinus MIT9313	0.205
Lactococcus lactis	Streptococcus agalactiae 2603	0.212
Pasteurella multocida	Escherichia coli 536	0.213
Pasteurella multocida	Salmonella enterica Choleraesuis	0.213
Haemophilus influenzae	Escherichia coli 536	0.216
Haemophilus ducreyi 35000HP	Escherichia coli 536	0.217
Haemophilus influenzae	Salmonella enterica Choleraesuis	0.217
Haemophilus ducreyi 35000HP	Salmonella enterica Choleraesuis	0.217
Bacillus subtilis	Bacillus halodurans	0.218
Prochlorococcus marinus MED4	Synechococcus sp WH8102	0.219
Lactococcus lactis	Streptococcus pyogenes MGAS315	0.219
Rhizobium leguminosarum bv viciae 3841	Brucella melitensis	0.219
Pasteurella multocida	Yersinia pestis biovar Mediaevails	0.219
Mycoplasma pneumoniae	Mycoplasma genitalium	0.222
Haemophilus influenzae	Yersinia pestis biovar Mediaevails	0.223
Haemophilus ducreyi 35000HP	Yersinia pestis biovar Mediaevails	0.223
Chromobacterium violaceum	Neisseria meningitidis FAM18	0.224
Rhizobium leguminosarum bv viciae 3841	Rhizobium etli CFN 42	0.224
Lactococcus lactis	Streptococcus mutans	0.224
Clostridium tetani E88	Clostridium perfringens	0.225
Agrobacterium tumefaciens C58 UWash	Brucella melitensis	0.225
Agrobacterium tumefaciens C58 UWash	Rhizobium etli CFN 42	0.230
Bacillus halodurans	Oceanobacillus iheyensis	0.231
Bacillus anthracis Ames	Bacillus halodurans	0.232
Chlamydia muridarum	Chlamydophila pneumoniae J138	0.234
Pasteurella multocida	Photorhabdus luminescens	0.236
Vibrio vulnificus YJ016	Escherichia coli 536	0.237
Vibrio vulnificus YJ016	Salmonella enterica Choleraesuis	0.238
Bacillus cereus ATCC14579	Bacillus halodurans	0.238
Haemophilus influenzae	Photorhabdus luminescens	0.239

Haemophilus ducreyi 35000HP	Photorhabdus luminescens	0.239
Vibrio cholerae	Escherichia coli 536	0.240
Vibrio cholerae	Salmonella enterica Choleraesuis	0.240
Chlamydia trachomatis	Chlamydophila pneumoniae J138	0.241
Vibrio vulnificus YJ016	Yersinia pestis biovar Mediaevails	0.244
Photobacterium profundum SS9	Escherichia coli 536	0.244
Photobacterium profundum SS9	Salmonella enterica Choleraesuis	0.245
Vibrio parahaemolyticus	Escherichia coli 536	0.245
Vibrio parahaemolyticus	Salmonella enterica Choleraesuis	0.245
Vibrio cholerae	Yersinia pestis biovar Mediaevails	0.246

List of genomes

Genome	Num. proteins	Num. domains	Num. families
Acaryochloris marina MBIC11017	8383	6583	1573
Acholeplasma laidlawii PG 8A	1380	1371	793
Acidiphilium cryptum JF-5	3559	3662	1486
Acidobacteria bacterium Ellin345	4777	4480	1483
Acidothermus cellulolyticus 11B	2157	2377	1176
Acidovorax JS42	4155	4126	1583
Acidovorax avenae citrulli AAC00-1	4709	4632	1646
Acinetobacter baumannii	3712	3647	1500
Acinetobacter baumannii ATCC 17978	3368	3087	1369
Acinetobacter baumannii AYE	3712	3647	1500
Acinetobacter baumannii SDF	2975	2926	1374
Acinetobacter sp ADP1	3325	3338	1458
Actinobacillus pleuropneumoniae L20	2012	2427	1358
Actinobacillus pleuropneumoniae serovar 3 JL03	2036	2363	1334
Actinobacillus succinogenes 130Z	2079	2575	1378
Aeromonas hydrophila ATCC 7966	4122	4768	1803
Aeromonas salmonicida A449	4437	4590	1814
Agrobacterium tumefaciens C58 Cereon	5355	5605	1721
Agrobacterium tumefaciens C58 UWash	5402	5609	1736
Alcanivorax borkumensis SK2	2755	2993	1415
Alkalilimnicola ehrlichei MLHE-1	2865	3222	1479
Alkaliphilus metalliredigens QYMF	4625	4341	1511
Alkaliphilus oremlandii OhILAs	2836	3054	1252
Anabaena variabilis ATCC 29413	5661	5776	1555
Anaeromyxobacter Fw109-5	4466	4492	1486
Anaeromyxobacter dehalogenans 2CP-C	4346	4334	1476
Anaplasma marginale St Maries	949	974	706
Anaplasma phagocytophilum HZ	1264	959	669
Aquifex aeolicus	1560	1785	1049
Arcobacter butzleri RM4018	2259	2297	1146
Arthrobacter FB24	4506	4440	1434
Arthrobacter aurescens TC1	4587	4405	1411
Aster yellows witches-broom phytoplasma AYWB	693	527	347
Azoarcus BH72	3989	4495	1627
Azoarcus sp EbN1	4599	4202	1517
Azorhizobium caulinodans ORS 571	4717	5075	1665
Bacillus amyloliquefaciens FZB42	3693	4159	1590
Bacillus anthracis A2012	5852	4865	1626

Bacillus anthracis Ames	5311	4802	1630
Bacillus anthracis Ames 0581	5617	4896	1650
Bacillus anthracis str Sterne	5287	5015	1646
Bacillus cereus ATCC14579	5255	4964	1635
Bacillus cereus ATCC 10987	5844	5108	1656
Bacillus cereus ZK	5641	5424	1659
Bacillus cereus cytotoxis NVH 391-98	3844	3706	1544
Bacillus clausii KSM-K16	4096	4349	1601
Bacillus halodurans	4066	4208	1627
Bacillus licheniformis ATCC 14580	4178	4249	1671
Bacillus licheniformis DSM 13	4196	4300	1679
Bacillus pumilus SAFR-032	3681	3821	1568
Bacillus subtilis	4105	4297	1655
Bacillus thuringiensis Al Hakam	4798	5037	1604
Bacillus thuringiensis konkukian	5197	5047	1636
Bacillus weihenstephanensis KBAB4	5653	5242	1655
Bacteroides fragilis NCTC 9434	4231	3641	1265
Bacteroides fragilis YCH46	4625	3698	1286
Bacteroides thetaiotaomicron VPI-5482	4816	4312	1308
Bacteroides vulgatus ATCC 8482	4065	3466	1203
Bartonella bacilliformis KC583	1283	1402	890
Bartonella henselae Houston-1	1488	1545	966
Bartonella quintana Toulouse	1142	1373	900
Bartonella tribocorum CIP 105476	2092	1801	993
Baumannia cicadellinicola Homalodisca coagulata	595	855	649
Bdellovibrio bacteriovorus	3587	2776	1219
Beijerinckia indica ATCC 9039	3784	3758	1528
Bifidobacterium adolescentis ATCC 15703	1631	1711	923
Bifidobacterium longum	1729	1816	969
Blochmannia floridanus	583	822	643
Bordetella bronchiseptica	4994	5541	1660
Bordetella parapertussis	4185	4772	1586
Bordetella pertussis	3436	3960	1471
Bordetella petrii	5027	5334	1653
Borrelia afzelii PKo	1214	1031	643
Borrelia burgdorferi	1640	1303	653
Borrelia garinii PBi	932	918	615
Bradyrhizobium BTAi1	7622	7332	1828
Bradyrhizobium ORS278	6717	6373	1779
Bradyrhizobium japonicum	8317	7740	1826
Brucella abortus 9-941	3085	3164	1460
Brucella canis ATCC 23365	3251	3259	1481
Brucella melitensis	3198	3309	1489
Brucella melitensis biovar Abortus	3034	3147	1454
Brucella ovis	2890	3056	1452
Brucella suis 1330	3271	3250	1481
Brucella suis ATCC 23445	3241	3212	1468
Buchnera aphidicola	507	739	572
Buchnera aphidicola Cc Cinara cedri	357	514	410
Buchnera aphidicola Sg	546	792	610
Buchnera sp	574	844	638
Burkholderia 383	7717	8023	1882
Burkholderia ambifaria MC40 6	6697	6980	1841
Burkholderia cenocepacia AU 1054	6477	6873	1848
Burkholderia cenocepacia HI2424	6919	7221	1870
Burkholderia cenocepacia MC0 3	7008	7539	1873
Burkholderia cepacia AMMD	6617	7068	1843

Burkholderia mallei ATCC 23344	5024	5066	1688
Burkholderia mallei NCTC 10229	5510	5132	1706
Burkholderia mallei NCTC 10247	5852	5260	1719
Burkholderia mallei SAVP1	5189	4772	1657
Burkholderia multivorans ATCC 17616	6259	6524	1843
Burkholderia pseudomallei 1106a	7183	6080	1814
Burkholderia pseudomallei 1710b	6347	6110	1822
Burkholderia pseudomallei 668	7230	6048	1809
Burkholderia pseudomallei K96243	5728	6179	1848
Burkholderia thailandensis E264	5634	5974	1838
Burkholderia vietnamiensis G4	7617	6967	1901
Burkholderia xenovorans LB400	8702	8720	1887
Caldicellulosiruptor saccharolyticus DSM 8903	2679	2764	1236
Campylobacter concisus 13826	1985	1662	962
Campylobacter curvus 525 92	1931	1797	1009
Campylobacter fetus 82-40	1719	1744	1033
Campylobacter hominis ATCC BAA-381	1687	1469	922
Campylobacter jejuni	1634	1666	1030
Campylobacter jejuni 81-176	1758	1692	1044
Campylobacter jejuni 81116	1626	1681	1041
Campylobacter jejuni RM1221	1838	1670	1045
Campylobacter jejuni doylei 269 97	1731	1605	996
Candidatus Blochmannia floridanus	583	822	643
Candidatus Blochmannia pennsylvanicus BPEN	610	860	664
Candidatus Desulfococcus oleovorans Hxd3	3265	3490	1254
Candidatus Desulforudis audaxviator MP104C	2157	2335	1177
Candidatus Pelagibacter ubique HTCC1062	1354	1448	905
Candidatus Ruthia magnifica Cm Calyptogena magnifica	976	1218	834
Candidatus Vesicomyosocius okutanii HA	937	1171	808
Carboxydothermus hydrogenoformans Z-2901	2620	2626	1296
Caulobacter K31	5438	5331	1680
Caulobacter crescentus	3737	3843	1550
Chlamydia muridarum	911	975	670
Chlamydia trachomatis	895	970	672
Chlamydia trachomatis 434 Bu	874	964	667
Chlamydia trachomatis A HAR-13	919	974	671
Chlamydia trachomatis L2b UCH 1 proctitis	874	965	668
Chlamydophila abortus S26 3	932	1002	679
Chlamydophila caviae	1005	1029	691
Chlamydophila felis Fe C-56	1013	1043	696
Chlamydophila pneumoniae AR39	1112	1040	686
Chlamydophila pneumoniae CWL029	1052	1044	687
Chlamydophila pneumoniae J138	1069	1049	687
Chlamydophila pneumoniae TW 183	1113	1048	689
Chlorobium chlorochromatii CaD3	2002	2100	1081
Chlorobium phaeobacteroides DSM 266	2650	2588	1186
Chlorobium tepidum TLS	2252	2077	1110
Chloroflexus aurantiacus J 10 fl	3853	4066	1383
Chromobacterium violaceum	4407	4570	1683
Chromohalobacter salexigens DSM 3043	3298	3812	1608
Citrobacter koseri ATCC BAA-895	5026	5108	1955
Clavibacter michiganensis NCPPB 382	3079	2922	1216
Clavibacter michiganensis sepedonicus	2941	2754	1173
Clostridium acetobutylicum	3848	3933	1428
Clostridium beijerinckii NCIMB 8052	5020	5444	1527
Clostridium botulinum A	3590	3597	1389

Clostridium botulinum A3 Loch Maree	3984	3769	1386
Clostridium botulinum A ATCC 19397	3552	3573	1379
Clostridium botulinum A Hall	3404	3497	1360
Clostridium botulinum B1 Okra	3852	3699	1397
Clostridium botulinum F Langeland	3659	3651	1383
Clostridium difficile 630	3753	4026	1384
Clostridium kluyveri DSM 555	3913	3648	1375
Clostridium novyi NT	2315	2503	1236
Clostridium perfringens	2723	2962	1317
Clostridium perfringens ATCC 13124	2876	3084	1332
Clostridium perfringens SM101	2631	2754	1261
Clostridium phytofermentans ISDg	3902	4014	1367
Clostridium tetani E88	2432	2710	1192
Clostridium thermocellum ATCC 27405	3189	3456	1322
Colwellia psychrerythraea 34H	4910	4575	1658
Corynebacterium diphtheriae	2272	2148	1124
Corynebacterium efficiens YS-314	2950	2682	1240
Corynebacterium glutamicum	2993	2716	1241
Corynebacterium glutamicum ATCC 13032 Bielefeld	3057	2723	1244
Corynebacterium glutamicum ATCC 13032 Kitasato	2993	2716	1241
Corynebacterium glutamicum R	3080	2833	1255
Corynebacterium jeikeium K411	2120	2111	1086
Corynebacterium urealyticum DSM 7109	2024	1989	1060
Coxiella burnetii	2052	1764	1013
Coxiella burnetii Dugway 7E9-12	2125	1821	1053
Coxiella burnetii RSA 331	1975	1722	995
Cyanobacteria bacterium Yellowstone A-Prime	2760	2695	1260
Cyanobacteria bacterium Yellowstone B-Prime	2862	2802	1285
Cyanothece ATCC 51142	5304	4442	1485
Cytophaga hutchinsonii ATCC 33406	3785	3121	1291
Dechloromonas aromatica RCB	4171	4616	1619
Dehalococcoides BAV1	1371	1441	812
Dehalococcoides CBDB1	1458	1528	824
Dehalococcoides ethenogenes 195	1580	1562	830
Deinococcus geothermalis DSM 11300	3062	2991	1300
Deinococcus radiodurans	3181	2935	1323
Delftia acidovorans SPH-1	6040	6167	1752
Desulfitobacterium hafniense Y51	5060	5207	1487
Desulfotalea psychrophila LSv54	3234	3150	1311
Desulfotomaculum reducens MI-1	3276	3390	1368
Desulfovibrio desulfuricans G20	3775	3499	1331
Desulfovibrio vulgaris DP4	3091	3369	1303
Desulfovibrio vulgaris Hildenborough	3531	3362	1307
Dichelobacter nodosus VCS1703A	1280	1424	948
Dinoroseobacter shibae DFL 12	4187	4110	1561
Ehrlichia canis Jake	925	942	680
Ehrlichia chaffeensis Arkansas	1105	953	683
Ehrlichia ruminantium Gardel	950	938	677
Ehrlichia ruminantium Welgevonden	888	933	677
Enterobacter 638	4240	4884	1958
Enterobacter sakazakii ATCC BAA-894	4434	4590	1926
Enterococcus faecalis V583	3265	3042	1307
Erwinia carotovora atroseptica SCRI1043	4472	4988	1917
Erythrobacter litoralis HTCC2594	3011	2756	1326
Escherichia coli 536	4629	5245	2013
Escherichia coli APEC O1	4880	5140	1964
Escherichia coli CFT073	5379	5438	2038

Escherichia coli DH10B	4126	4905	1961
Escherichia coli E24377A	4997	5344	2060
Escherichia coli HS	4384	4979	2017
Escherichia coli K12	4133	4992	2009
Escherichia coli K 12 substr DH10B	4126	4905	1961
Escherichia coli K 12 substr MG1655	4132	4991	2008
Escherichia coli O157H7	5341	5741	2149
Escherichia coli O157H7 EDL933	5423	5742	2127
Escherichia coli SECEC SMS 3 5	4913	5415	2071
Escherichia coli UTI89	5189	5443	2112
Escherichia coli W3110	4226	5039	2012
Exiguobacterium sibiricum 255 15	3015	3117	1374
Fervidobacterium nodosum Rt17-B1	1750	1872	1020
Finegoldia magna ATCC 29328	1813	1898	971
Flavobacterium johnsoniae UW101	5017	4202	1432
Flavobacterium psychrophilum JIP02 86	2412	2075	1089
Francisella philomiragia ATCC 25017	1915	1968	1104
Francisella tularensis FSC 198	1605	1588	984
Francisella tularensis WY96-3418	1634	1614	1004
Francisella tularensis holarctica	1754	1628	997
Francisella tularensis holarctica FTA	1580	1550	968
Francisella tularensis holarctica OSU18	1555	1528	971
Francisella tularensis novicida U112	1719	1801	1062
Francisella tularensis tularensis	1603	1583	983
Frankia CcI3	4499	3905	1344
Frankia EAN1pec	7191	6342	1465
Frankia alni ACN14a	6711	5491	1418
Fusobacterium nucleatum	2067	1963	1059
Geobacillus kaustophilus HTA426	3540	3523	1563
Geobacillus thermodenitrificans NG80-2	3445	3533	1558
Geobacter metallireducens GS-15	3532	3977	1408
Geobacter sulfurreducens	3446	3785	1369
Geobacter uraniumreducens Rf4	4357	4661	1436
Gloeobacter violaceus	4430	4282	1400
Gluconacetobacter diazotrophicus PAl 5	3852	3539	1451
Gluconobacter oxydans 621H	2664	2537	1266
Gramella forsetii KT0803	3584	3029	1286
Granulobacter bethesdensis CGDNIH1	2437	2520	1325
Haemophilus ducreyi 35000HP	1717	1695	1093
Haemophilus influenzae	1657	2047	1242
Haemophilus influenzae 86 028NP	1792	2067	1254
Haemophilus influenzae PittEE	1619	1903	1174
Haemophilus influenzae PittGG	1667	1845	1146
Haemophilus somnus 129PT	1798	2107	1210
Haemophilus somnus 2336	1980	2245	1273
Hahella chejuensis KCTC 2396	6778	5881	1829
Halorhodospira halophila SL1	2407	2708	1352
Helicobacter acinonychis Sheeba	1618	1438	897
Helicobacter hepaticus	1875	1643	1004
Helicobacter pylori 26695	1576	1561	934
Helicobacter pylori HPAG1	1544	1559	936
Helicobacter pylori J99	1489	1577	941
Heliobacterium modesticaldum Ice1	3000	2873	1288
Herminiimonas arsenicoxydans	3325	3287	1485
Herpetosiphon aurantiacus ATCC 23779	5278	5038	1433
Hyphomonas neptunium ATCC 15444	3505	3444	1466
Idiomarina loihiensis L2TR	2628	2960	1428

Jannaschia CCS1	4283	4111	1546
Janthinobacterium Marseille	3697	3977	1548
Kineococcus radiotolerans SRS30216	4681	4374	1399
Klebsiella pneumoniae MGH 78578	5187	5842	2020
Lactobacillus acidophilus NCFM	1862	1950	933
Lactobacillus brevis ATCC 367	2218	2128	1035
Lactobacillus casei ATCC 334	2771	2605	1146
Lactobacillus delbrueckii bulgaricus	1562	1540	860
Lactobacillus delbrueckii bulgaricus ATCC BAA-365	1721	1570	876
Lactobacillus gasseri ATCC 33323	1755	1848	922
Lactobacillus helveticus DPC 4571	1610	1728	905
Lactobacillus johnsonii NCC 533	1821	1971	936
Lactobacillus plantarum	3057	3093	1235
Lactobacillus reuteri F275	1900	1943	1036
Lactobacillus sakei 23K	1879	1911	1020
Lactobacillus salivarius UCC118	2017	1960	1047
Lactococcus lactis	2321	2348	1156
Lactococcus lactis cremoris MG1363	2434	2377	1154
Lactococcus lactis cremoris SK11	2504	2408	1131
Lawsonia intracellularis PHE MN1-00	1337	1444	887
Legionella pneumophila Corby	3206	2785	1338
Legionella pneumophila Lens	2934	2711	1341
Legionella pneumophila Paris	3166	2860	1360
Legionella pneumophila Philadelphia 1	2942	2742	1322
Leifsonia xyli xyli CTCB0	2030	1908	1009
Leptospira biflexa serovar Patoc Patoc 1 Paris	3391	2922	1175
Leptospira borgpetersenii serovar Hardjo-bovis JB197	2880	2610	1152
Leptospira borgpetersenii serovar Hardjo-bovis L550	2945	2679	1154
Leptospira interrogans serovar Copenhageni	3658	2917	1211
Leptospira interrogans serovar Lai	4727	3101	1215
Leptothrix cholodnii SP 6	4363	4826	1677
Leuconostoc citreum KM20	1820	1891	997
Leuconostoc mesenteroides ATCC 8293	2005	2040	1039
Listeria innocua	3043	3260	1419
Listeria monocytogenes	2846	3209	1405
Listeria monocytogenes 4b F2365	2821	3143	1376
Listeria welshimeri serovar 6b SLCC5334	2774	3041	1359
Lysinibacillus sphaericus C3 41	4771	4203	1564
Magnetococcus MC-1	3716	3922	1341
Magnetospirillum magneticum AMB-1	4559	4356	1470
Mannheimia succiniciproducens MBEL55E	2380	2559	1362
Maricaulis maris MCS10	3063	3085	1438
Marinobacter aquaeolei VT8	4272	4277	1621
Marinomonas MWYL1	4439	5011	1681
Mesoplasma florum L1	682	721	468
Mesorhizobium BNC1	4543	4676	1600
Mesorhizobium loti	7272	7049	1838
Methylibium petroleiphilum PM1	4449	4263	1632
Methylobacillus flagellatus KT	2753	3043	1376
Methylobacterium 4 46	6692	6276	1758
Methylobacterium extorquens PA1	4829	4630	1628
Methylobacterium radiotolerans JCM 2831	6431	5922	1715
Methylococcus capsulatus Bath	2956	3023	1428
Microcystis aeruginosa NIES 843	6312	4262	1394
Moorella thermoacetica ATCC 39073	2465	2756	1303
Mycobacterium JLS	5739	5470	1484
Mycobacterium KMS	5975	5452	1493

Mycobacterium MCS	5615	5239	1482
Mycobacterium abscessus ATCC 19977T	4941	4379	1381
Mycobacterium avium 104	5120	4684	1362
Mycobacterium avium paratuberculosis	4350	4297	1335
Mycobacterium bovis	3920	3973	1352
Mycobacterium bovis BCG Pasteur 1173P2	3952	4011	1344
Mycobacterium gilvum PYR-GCK	5579	5172	1491
Mycobacterium leprae	1605	1676	950
Mycobacterium marinum M	5452	5669	1444
Mycobacterium smegmatis MC2 155	6716	6316	1537
Mycobacterium tuberculosis CDC1551	4189	3912	1339
Mycobacterium tuberculosis F11	3941	4076	1349
Mycobacterium tuberculosis H37Ra	4034	4085	1353
Mycobacterium tuberculosis H37Rv	3989	4079	1354
Mycobacterium ulcerans Agy99	4160	4011	1308
Mycobacterium vanbaalenii PYR-1	5979	5612	1517
Mycoplasma agalactiae PG2	742	630	431
Mycoplasma capricolum ATCC 27343	812	766	494
Mycoplasma gallisepticum	726	727	450
Mycoplasma genitalium	477	561	418
Mycoplasma hyopneumoniae 232	691	602	410
Mycoplasma hyopneumoniae 7448	663	610	415
Mycoplasma hyopneumoniae J	665	604	413
Mycoplasma mobile 163K	633	658	440
Mycoplasma mycoides	1016	828	488
Mycoplasma penetrans	1037	925	533
Mycoplasma pneumoniae	689	717	438
Mycoplasma pulmonis	782	744	470
Mycoplasma synoviae 53	672	630	424
Myxococcus xanthus DK 1622	7331	6541	1654
Neisseria gonorrhoeae FA 1090	2002	1844	1162
Neisseria meningitidis 053442	2020	1958	1206
Neisseria meningitidis FAM18	1917	2026	1220
Neisseria meningitidis MC58	2063	2050	1221
Neisseria meningitidis Z2491	2049	1982	1221
Neorickettsia sennetsu Miyayama	932	805	613
Nitratiruptor SB155-2	1843	1949	1071
Nitrobacter hamburgensis X14	4326	3715	1528
Nitrobacter winogradskyi Nb-255	3122	2892	1381
Nitrosococcus oceani ATCC 19707	3017	3013	1412
Nitrosomonas europaea	2461	2670	1305
Nitrosomonas eutropha C71	2551	2595	1315
Nitrosospira multiformis ATCC 25196	2805	2840	1348
Nocardia farcinica IFM10152	5936	5155	1489
Nocardioides JS614	4909	4660	1455
Nostoc sp	6130	5798	1553
Novosphingobium aromaticivorans DSM 12444	3937	3922	1490
Oceanobacillus iheyensis	3500	3565	1496
Ochrobactrum anthropi ATCC 49188	4799	4984	1696
Oenococcus oeni PSU-1	1691	1711	903
Onion yellows phytoplasma	754	599	351
Opitutus terrae PB90 1	4612	4640	1418
Orientia tsutsugamushi Boryong	1182	1028	589
Parabacteroides distasonis ATCC 8503	3850	3557	1265
Parachlamydia sp UWE25	2031	1535	907
Paracoccus denitrificans PD1222	5077	5143	1667
Parvibaculum lavamentivorans DS-1	3636	3703	1485

Pasteurella multocida	2015	2451	1350
Pediococcus pentosaceus ATCC 25745	1755	1912	1002
Pelobacter carbinolicus	3352	3502	1380
Pelobacter propionicus DSM 2379	3804	3911	1379
Pelodictyon luteolum DSM 273	2083	2270	1137
Pelotomaculum thermopropionicum SI	2920	2910	1262
Petrotoga mobilis SJ95	1898	2096	1093
Photobacterium profundum SS9	5489	5338	1858
Photorhabdus luminescens	4683	4450	1778
Pirellula sp	7325	4760	1428
Polaromonas JS666	5453	5422	1620
Polaromonas naphthalenivorans CJ2	4929	4610	1672
Polynucleobacter QLW-P1DMWA-1	2077	2257	1227
Polynucleobacter necessarius STIR1	1508	1467	979
Porphyromonas gingivalis W83	1909	1729	964
Prochlorococcus marinus AS9601	1921	1623	992
Prochlorococcus marinus CCMP1375	1883	1632	1000
Prochlorococcus marinus MED4	1717	1600	998
Prochlorococcus marinus MIT9313	2269	2050	1110
Prochlorococcus marinus MIT 9211	1855	1625	996
Prochlorococcus marinus MIT 9215	1983	1603	990
Prochlorococcus marinus MIT 9301	1907	1608	996
Prochlorococcus marinus MIT 9303	2997	2266	1133
Prochlorococcus marinus MIT 9312	1810	1611	1002
Prochlorococcus marinus MIT 9515	1906	1577	980
Prochlorococcus marinus NATL1A	2193	1670	1006
Prochlorococcus marinus NATL2A	2163	1685	1001
Propionibacterium acnes KPA171202	2297	2250	1124
Prosthecochloris vibrioformis DSM 265	1753	1987	1083
Pseudoalteromonas atlantica T6c	4281	4568	1715
Pseudoalteromonas haloplanktis TAC125	3486	3633	1610
Pseudomonas aeruginosa	5568	6330	1933
Pseudomonas aeruginosa PA7	6286	6363	1982
Pseudomonas aeruginosa UCBPP-PA14	5892	6431	1966
Pseudomonas entomophila L48	5134	5628	1843
Pseudomonas fluorescens Pf-5	6138	6829	1927
Pseudomonas fluorescens PfO-1	5736	6261	1888
Pseudomonas mendocina ymp	4594	5135	1775
Pseudomonas putida F1	5252	5861	1861
Pseudomonas putida GB 1	5409	5986	1861
Pseudomonas putida KT2440	5350	5871	1853
Pseudomonas putida W619	5182	5693	1855
Pseudomonas stutzeri A1501	4128	4593	1747
Pseudomonas syringae	5607	5812	1875
Pseudomonas syringae phaseolicola 1448A	5171	5587	1836
Pseudomonas syringae pv B728a	5089	5714	1857
Pseudomonas syringae tomato DC3000	5613	5812	1875
Psychrobacter PRwf-1	2385	2546	1291
Psychrobacter arcticum 273-4	2120	2245	1254
Psychrobacter cryohalolentis K5	2511	2707	1349
Psychromonas ingrahamii 37	3545	3782	1611
Ralstonia eutropha H16	6626	6700	1765
Ralstonia eutropha JMP134	6446	6928	1802
Ralstonia metallidurans CH34	6319	6343	1770
Ralstonia solanacearum	5116	5213	1756
Renibacterium salmoninarum ATCC 33209	3507	2824	1199
Rhizobium etli CFN 42	5963	5873	1740

Rhizobium leguminosarum bv viciae 3841	7143	7321	1822
Rhodobacter sphaeroides 2 4 1	4242	4356	1656
Rhodobacter sphaeroides ATCC 17025	4333	4194	1621
Rhodobacter sphaeroides ATCC 17029	4132	4324	1636
Rhodococcus RHA1	9145	8184	1604
Rhodoferax ferrireducens DSM 15236	4418	4705	1615
Rhodoferax ferrireducens T118	4418	4705	1615
Rhodopseudomonas palustris BisA53	4878	4688	1676
Rhodopseudomonas palustris BisB18	4886	4847	1686
Rhodopseudomonas palustris BisB5	4397	4387	1624
Rhodopseudomonas palustris CGA009	4820	4950	1677
Rhodopseudomonas palustris HaA2	4683	4874	1659
Rhodospirillum rubrum ATCC 11170	3841	4155	1571
Rickettsia akari Hartford	1259	993	708
Rickettsia bellii OSU 85-389	1476	1215	772
Rickettsia bellii RML369-C	1429	1237	786
Rickettsia canadensis McKiel	1093	963	689
Rickettsia conorii	1374	1043	733
Rickettsia felis URRWXCal2	1512	1306	797
Rickettsia massiliae MTU5	980	1067	730
Rickettsia prowazekii	835	978	697
Rickettsia rickettsii Iowa	1384	1022	720
Rickettsia rickettsii Sheila Smith	1345	1016	723
Rickettsia typhi wilmington	838	969	689
Roseiflexus RS-1	4517	4581	1405
Roseiflexus castenholzii DSM 13941	4330	4316	1401
Roseobacter denitrificans OCh 114	4129	4072	1540
Rubrobacter xylanophilus DSM 9941	3140	3355	1271
Saccharophagus degradans 2-40	4008	4082	1612
Saccharopolyspora erythraea NRRL 2338	7197	6814	1633
Salinibacter ruber DSM 13855	2833	2897	1225
Salinispora arenicola CNS-205	4917	4627	1437
Salinispora tropica CNB-440	4536	4293	1395
Salmonella enterica Choleraesuis	4648	5005	2024
Salmonella enterica Paratypi ATCC 9150	4093	4684	1968
Salmonella enterica arizonae serovar 62 z4 z23	4510	4585	1950
Salmonella enterica serovar Paratyphi B SPB7	5601	5045	2004
Salmonella typhi	4758	4966	2034
Salmonella typhi Ty2	4318	4802	1984
Salmonella typhimurium LT2	4527	5236	2074
Serratia proteamaculans 568	4942	5642	2019
Shewanella ANA-3	4360	4549	1767
Shewanella MR-4	3924	4286	1723
Shewanella MR-7	4014	4278	1743
Shewanella W3-18-1	4044	4217	1751
Shewanella amazonensis SB2B	3645	4081	1666
Shewanella baltica OS155	4489	4685	1750
Shewanella baltica OS185	4394	4538	1782
Shewanella baltica OS195	4688	4687	1807
Shewanella denitrificans OS217	3754	3916	1627
Shewanella frigidimarina NCIMB 400	4029	4326	1708
Shewanella halifaxensis HAW EB4	4278	4467	1708
Shewanella loihica PV-4	3859	4145	1681
Shewanella oneidensis	4467	4406	1743
Shewanella pealeana ATCC 700345	4241	4383	1695
Shewanella putrefaciens CN-32	3972	4222	1724
Shewanella sediminis HAW-EB3	4497	4744	1694

Shewanella woodyi ATCC 51908	4880	4951	1796
Shigella boydii Sb227	4285	4748	1930
Shigella dysenteriae	4506	4808	1817
Shigella flexneri 2a	4445	4936	1920
Shigella flexneri 2a 2457T	4068	4658	1870
Shigella flexneri 5 8401	4116	4688	1887
Shigella sonnei Ss046	4475	5124	1992
Silicibacter TM1040	3864	4005	1557
Silicibacter pomeroyi DSS-3	4252	4487	1523
Sinorhizobium medicae WSM419	6213	6469	1780
Sinorhizobium meliloti	6205	6505	1801
Sodalis glossinidius morsitans	2516	2599	1424
Solibacter usitatus Ellin6076	7826	7200	1663
Sorangium cellulosum So ce 56	9384	7990	1673
Sphingomonas wittichii RW1	5345	5466	1569
Sphingopyxis alaskensis RB2256	3195	3211	1430
Staphylococcus aureus COL	2618	2882	1367
Staphylococcus aureus JH1	2780	3015	1383
Staphylococcus aureus JH9	2726	3019	1382
Staphylococcus aureus MW2	2632	2920	1375
Staphylococcus aureus Mu3	2698	2979	1382
Staphylococcus aureus Mu50	2731	2991	1387
Staphylococcus aureus N315	2619	2961	1367
Staphylococcus aureus NCTC 8325	2892	2891	1368
Staphylococcus aureus Newman	2614	2909	1349
Staphylococcus aureus RF122	2509	2657	1340
Staphylococcus aureus USA300	2604	2958	1383
Staphylococcus aureus USA300 TCH1516	2683	2817	1323
Staphylococcus aureus aureus MRSA252	2656	2912	1369
Staphylococcus aureus aureus MSSA476	2598	2782	1370
Staphylococcus epidermidis ATCC 12228	2485	2625	1274
Staphylococcus epidermidis RP62A	2526	2646	1280
Staphylococcus haemolyticus	2676	2658	1319
Staphylococcus saprophyticus	2514	2613	1292
Streptococcus agalactiae 2603	2124	2213	1119
Streptococcus agalactiae A909	1996	2203	1115
Streptococcus agalactiae NEM316	2094	2182	1091
Streptococcus gordonii Challis substr CH1	2051	2203	1113
Streptococcus mutans	1960	2076	1078
Streptococcus pneumoniae CGSP14	2206	2284	1138
Streptococcus pneumoniae D39	1914	2167	1099
Streptococcus pneumoniae Hungary19A 6	2155	2246	1143
Streptococcus pneumoniae R6	2043	2212	1118
Streptococcus pneumoniae TIGR4	2105	2237	1109
Streptococcus pyogenes	1697	1870	1049
Streptococcus pyogenes M1 GAS	1697	1870	1049
Streptococcus pyogenes MGAS10270	1986	1960	1069
Streptococcus pyogenes MGAS10394	1886	1912	1044
Streptococcus pyogenes MGAS10750	1979	1965	1067
Streptococcus pyogenes MGAS2096	1898	1893	1031
Streptococcus pyogenes MGAS315	1865	1919	1058
Streptococcus pyogenes MGAS5005	1865	1890	1052
Streptococcus pyogenes MGAS6180	1894	1953	1059
Streptococcus pyogenes MGAS8232	1839	1906	1054
Streptococcus pyogenes MGAS9429	1877	1909	1046
Streptococcus pyogenes Manfredo	1745	1853	1045
Streptococcus pyogenes SSI-1	1861	1891	1053

Streptococcus sanguinis SK36	2270	2405	1168
Streptococcus suis 05ZYH33	2186	2067	1102
Streptococcus suis 98HAH33	2185	2081	1100
Streptococcus thermophilus CNRZ1066	1915	1781	1023
Streptococcus thermophilus LMD-9	1716	1711	1006
Streptococcus thermophilus LMG 18311	1889	1772	1024
Streptomyces avermitilis	7676	7305	1643
Streptomyces coelicolor	8154	7691	1689
Streptomyces griseus NBRC 13350	7136	6948	1623
Sulfurovum NBC37-1	2438	2316	1152
Symbiobacterium thermophilum IAM14863	3338	3350	1373
Synechococcus CC9311	2892	2196	1191
Synechococcus CC9605	2645	2082	1159
Synechococcus CC9902	2307	2015	1149
Synechococcus PCC 7002	3186	3029	1360
Synechococcus RCC307	2535	2137	1180
Synechococcus WH 7803	2533	2154	1175
Synechococcus elongatus PCC 6301	2527	2577	1294
Synechococcus elongatus PCC 7942	2729	2673	1312
Synechococcus sp WH8102	2519	2124	1157
Synechocystis PCC6803	3569	3502	1415
Syntrophobacter fumaroxidans MPOB	4064	4108	1370
Syntrophomonas wolfei Goettingen	2504	2680	1207
Syntrophus aciditrophicus SB	3168	2971	1256
Thermoanaerobacter X514	2349	2606	1288
Thermoanaerobacter pseudethanolicus ATCC 33223	2243	2524	1271
Thermoanaerobacter tengcongensis	2588	2763	1274
Thermobifida fusca YX	3110	3044	1266
Thermosipho melanesiensis BI429	1879	1905	1040
Thermosynechococcus elongatus	2476	2567	1225
Thermotoga lettingae TMO	2040	2302	1100
Thermotoga maritima	1858	2089	1119
Thermotoga petrophila RKU-1	1785	2061	1116
Thermus thermophilus HB27	2210	2292	1162
Thermus thermophilus HB8	2238	2285	1158
Thiobacillus denitrificans ATCC 25259	2827	2982	1394
Thiomicrospira crunogena XCL-2	2196	2455	1266
Thiomicrospira denitrificans ATCC 33889	2096	2150	1093
Treponema denticola ATCC 35405	2767	2196	1033
Treponema pallidum	1036	1053	660
Trichodesmium erythraeum IMS101	4451	4614	1389
Tropheryma whipplei TW08 27	783	949	627
Tropheryma whipplei Twist	808	948	633
Ureaplasma parvum serovar 3 ATCC 27815	609	573	411
Ureaplasma urealyticum	614	573	411
Verminephrobacter eiseniae EF01-2	4947	4985	1501
Vibrio cholerae	3835	4053	1717
Vibrio cholerae O395	3875	4137	1755
Vibrio fischeri ES114	3818	4077	1687
Vibrio harveyi ATCC BAA-1116	6055	5245	1857
Vibrio parahaemolyticus	4832	4892	1815
Vibrio vulnificus CMCP6	4484	4772	1796
Vibrio vulnificus YJ016	5024	4940	1814
Wigglesworthia brevipalpis	617	841	645
Wolbachia endosymbiont of Brugia malayi TRS	805	867	641
Wolbachia endosymbiont of Drosophila melanogaster	1195	1082	721
Wolinella succinogenes	2042	2258	1093

Xanthobacter autotrophicus Py2	5035	4968	1656
Xanthomonas campestris	4181	4390	1725
Xanthomonas campestris 8004	4273	4386	1717
Xanthomonas campestris vesicatoria 85-10	4726	4594	1753
Xanthomonas citri	4427	4527	1761
Xanthomonas oryzae KACC10331	4144	3708	1578
Xanthomonas oryzae MAFF 311018	4372	3911	1613
Xylella fastidiosa	2832	2283	1255
Xylella fastidiosa M12	2104	2088	1210
Xylella fastidiosa M23	2201	2193	1245
Xylella fastidiosa Temecula1	2036	2140	1225
Yersinia enterocolitica 8081	4051	4657	1949
Yersinia pestis Angola	4045	4264	1805
Yersinia pestis Antiqua	4364	4753	1881
Yersinia pestis CO92	4066	4504	1862
Yersinia pestis KIM	4202	4423	1833
Yersinia pestis Nepal516	4094	4501	1854
Yersinia pestis Pestoides F	4069	4495	1886
Yersinia pestis biovar Mediaevails	4142	4495	1871
Yersinia pseudotuberculosis IP32953	4038	4589	1913
Yersinia pseudotuberculosis IP 31758	4324	4553	1898
Yersinia pseudotuberculosis YPIII	4192	4442	1879
Zymomonas mobilis ZM4	1998	1953	1087

Change in total domain count vs. number of domain-count changes

Given two closely-related genomes with domain counts n_c^1 and n_c^2 in category c, the change in the total domain-count is given by

$$dn_c = |n_c^1 - n_c^2|, \quad (1)$$

which should be clearly distinguished from the number of domain-count changes Δn_c. That is, Δn_c denotes the total number of domain additions and deletions that have occurred in the two lineages leading from the common ancestor of the two organisms. When the organisms are very closely related and when the number of domains $\bar{n}_c = (n_c^1 + n_c^2)/2$ is small, the number of changes Δn_c is often close to dn_c, but for more distant pairs and when $\bar{n}_c$ is large, we often have that Δn_c is significantly larger than dn_c. To illustrate this we have calculated, for each genome pair i and each category c, the ratio R_c^i of the change in the total number of domains dn_c^i and the estimated total number of domain-count changes Δn_c^i, i.e.

$$R_c^i = \frac{dn_c^i}{\Delta n_c^i}. \quad (2)$$

Figure 1 shows a histogram of the ratios R_c^i that we obtained. As Fig. 1 shows, about 20% of the r_c^i are close to 1, i.e. 0.9 or larger, meaning that the change in the total dn_c^i reasonably accurately reflects the total number of

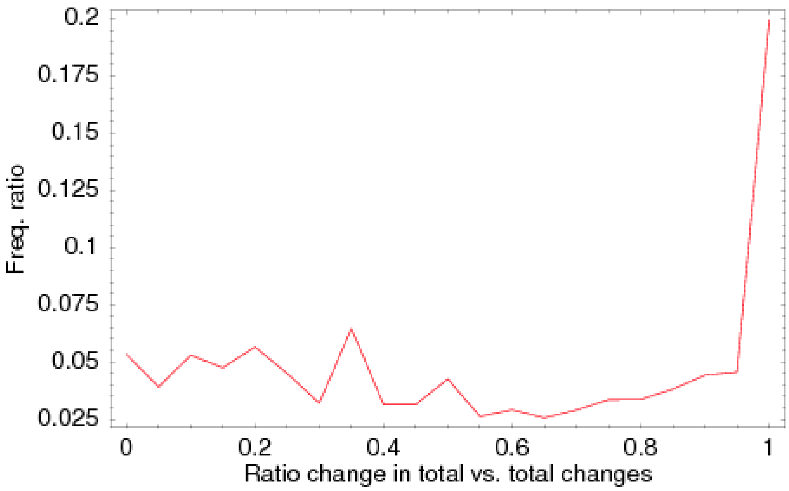

Figure 1: Histogram of the ratios r_c^i of the total change in domain-count in category c between the pair of related species i, and the total number of domain-count changes Δn_c^i that occurred in the two lineages since the pair of genomes separated from their common ancestor.

changes Δn_c^i. However, for the large majority of category/lineage combinations there is a significant difference between dn_c^i and Δn_c^i, and their ratio R_c^i is roughly uniformly distributed.
